# Evolutionarily conserved BIL4 suppresses the degradation of brassinosteroid receptor BRI1 and regulates cell elongation

**DOI:** 10.1038/s41598-017-06016-2

**Published:** 2017-07-18

**Authors:** Ayumi Yamagami, Chieko Saito, Miki Nakazawa, Shozo Fujioka, Tomohiro Uemura, Minami Matsui, Masaaki Sakuta, Kazuo Shinozaki, Hiroyuki Osada, Akihiko Nakano, Tadao Asami, Takeshi Nakano

**Affiliations:** 1 Gene Discovery Research Group, RIKEN Center for Sustainable Resource Science, Wako, Saitama 351-0198 Japan; 2 Molecular Membrane Biology Laboratory, RIKEN, Wako, Saitama 351-0198 Japan; 3 Department of Biological Sciences, Graduate School of Science, The University of Tokyo, Hongo, Bunkyo-ku, Tokyo 113-0033 Japan; 4 RIKEN Genomic Science Center, Tsurumi, Yokohama, Kanagawa 230-0045 Japan; 5 Synthetic Genomics Research Group, RIKEN Center for Sustainable Resource Science, Tsurumi, Yokohama, Kanagawa 230-0045 Japan; 6 Department of Biological Sciences, Ochanomizu University, Ohtsuka, Bunkyo-ku, Tokyo 112-8610 Japan; 7 Chemical Biology Research Group, RIKEN Center for Sustainable Resource Science, Wako, Saitama 351-0198 Japan; 8 Live Cell Super-Resolution Imaging Research Team, RIKEN Center for Advanced Photonics, Wako, Saitama 351-0198 Japan; 9 Department of Applied Biological Chemistry, The University of Tokyo, Yayoi, Bunkyo-ku, Tokyo 113-8657 Japan; 10 King Abdulaziz University, Department of Biochemistry, Jeddah, 21589 Saudi Arabia; 11 CREST, JST (Japan Science and Technology Agency), Kawaguchi, Saitama 332-0012 Japan

## Abstract

Brassinosteroids (BRs), plant steroid hormones, play important roles in plant cell elongation and differentiation. To investigate the mechanisms of BR signaling, we previously used the BR biosynthesis inhibitor Brz as a chemical biology tool and identified the *Brz-insensitive-long hypocotyl4* mutant (*bil4*). Although the *BIL4* gene encodes a seven-transmembrane-domain protein that is evolutionarily conserved in plants and animals, the molecular function of BIL4 in BR signaling has not been elucidated. Here, we demonstrate that BIL4 is expressed in early elongating cells and regulates cell elongation in *Arabidopsis*. BIL4 also activates BR signaling and interacts with the BR receptor brassinosteroid insensitive 1 (BRI1) in endosomes. BIL4 deficiency increases the localization of BRI1 in the vacuoles. Our results demonstrate that BIL4 regulates cell elongation and BR signaling via the regulation of BRI1 localization.

## Introduction

Steroid hormones are bioactive substances that are widely conserved in eukaryotes^1^ and serve as signaling molecules to control growth and development. The plant steroid hormones known as brassinosteroids (BRs) play important roles in plant development and in responses to environmental cues, such as cell elongation, cell division, xylem development, stresses and pathogen resistance^2–6^. BRs are recognized by the BR receptor brassinosteroid insensitive 1 (BRI1), a Ser/Thr kinase, which has a leucine-rich repeat (LRR) domain and resides on the plasma membrane and endosomes. The dwarf phenotype of BRI1-deficient mutants (*bri1*)^7^ suggests that BRI1 plays an important role in plant growth. The plasma membrane proteins BRI1-associated receptor kinase 1 (BAK1)^8, 9^, BR-signaling kinase 1 (BSK1)^10^, and BRI1 kinase inhibitor 1 (BKI1)^11^ are involved in BR signaling by associating with BRI1.

BRI1 is localized not only to plasma membranes but also to endosomes^12^. The requirement that ligand-bound receptors, including innate immunity-related Toll-like receptors (TLRs), be localized to endosomes has been widely observed in animals^13^. BRI1 endocytosis is regulated by the guanine nucleotide exchange factor for ADP-ribosylation factor (ARF-GEF) GNOM^14^, adaptor protein complex-2 (AP-2)^15^, and plant-specific TPLATE adaptor complex (TPC), which is associated with key components of clathrin-mediated endocytosis (CME)^16^. BRI1 is considered to translocate to the vacuoles through processes that are regulated by ubiquitination^17^ and ESCRT protein^18^, which mediates cargo protein transport to intraluminal vesicles (ILVs) of late endosome/multivesicular body (LE/MVB). However, the detailed molecular mechanisms of BRI1 endocytic trafficking remain to be fully elucidated.

We have previously synthesized Brz (brassinazole), a specific inhibitor of BR biosynthesis^19^. Through its triazole base, Brz directly binds to the DWF4 enzyme, a cytochrome P450 monooxygenase that catalyzes the 22-hydroxylation of the BR sidechains. Brz treatment decreases the BR content^20^ and causes a dwarf phenotype in plants, which is similar to the phenotype of BR-deficient mutants. These responses to Brz were used to search in a chemical biology screen for gain-of-function mutants that are resistant to Brz. The first gene identified using this approach was *BZR1* ^21^. *BIL1* has been identified to be identical to *BZR1* ^22^, and *BES1* is homologous to *BZR1* ^23^. The *BIL1*/*BZR1* and *BES1* genes encode master transcription factors that regulate the expression of thousands of genes^24–26^.

In our recent study, we have identified *Brz-insensitive-long hypocotyl 4–1D* (*bil4-1D*) from activation-tagging lines of *Arabidopsis thaliana* (hereafter, *Arabidopsis*) with a chemical biology approach using Brz^27^. The *bil4-1D* phenotype is caused by the overexpression of a novel gene. *BIL4* has homologous genes in other plant species (e.g., tomato, rice and maize). Hydropathy plot analysis suggests that BIL4 contains seven transmembrane domains. The hypocotyl phenotype of *bil4-1D* may suggest that BIL4 acts as a positive regulator in BR signaling, but the mechanism of action of BIL4 in BR signaling and plant development has not been predicted. In this study, we identified BIL4 to be a regulatory factor affecting both endocytosis of the BR receptor BRI1 and BR-mediated plant cell elongation.

## Results

### BIL4 positively regulates BR signaling

In the presence of Brz, dark-grown wild-type *Arabidopsis* seedlings exhibit de-etiolation traits, such as short hypocotyls and open cotyledons, as if the plants were grown under light^28^. Both *bil4-1D* and *BIL4-OX* plants exhibit an elongated hypocotyl when grown on Brz in the dark^27^. In this study, both *bil4-1D* and *BIL4* overexpressor (*BIL4-OX*) plants, as compared with the wild-type, in the adult stage showed slender dwarf leaves and an increased number of branches, whereas the petioles and leaf blades were slightly smaller than those of the wild-type (Supplementary Fig. S1). We have previously found that the rosette leaves of plants exposed to excessive concentrations of BR are smaller than those of mock-treated plants^27^. The hypocotyl elongation of *bil4-1D* and *BIL4-OX* reflects the increased BR signaling and suggests that *BIL4* may be a positive regulator of BR signaling^27^. However, the detailed role and importance of BIL4 in plant development and BR signaling have not yet been analyzed.

**Figure 1:**
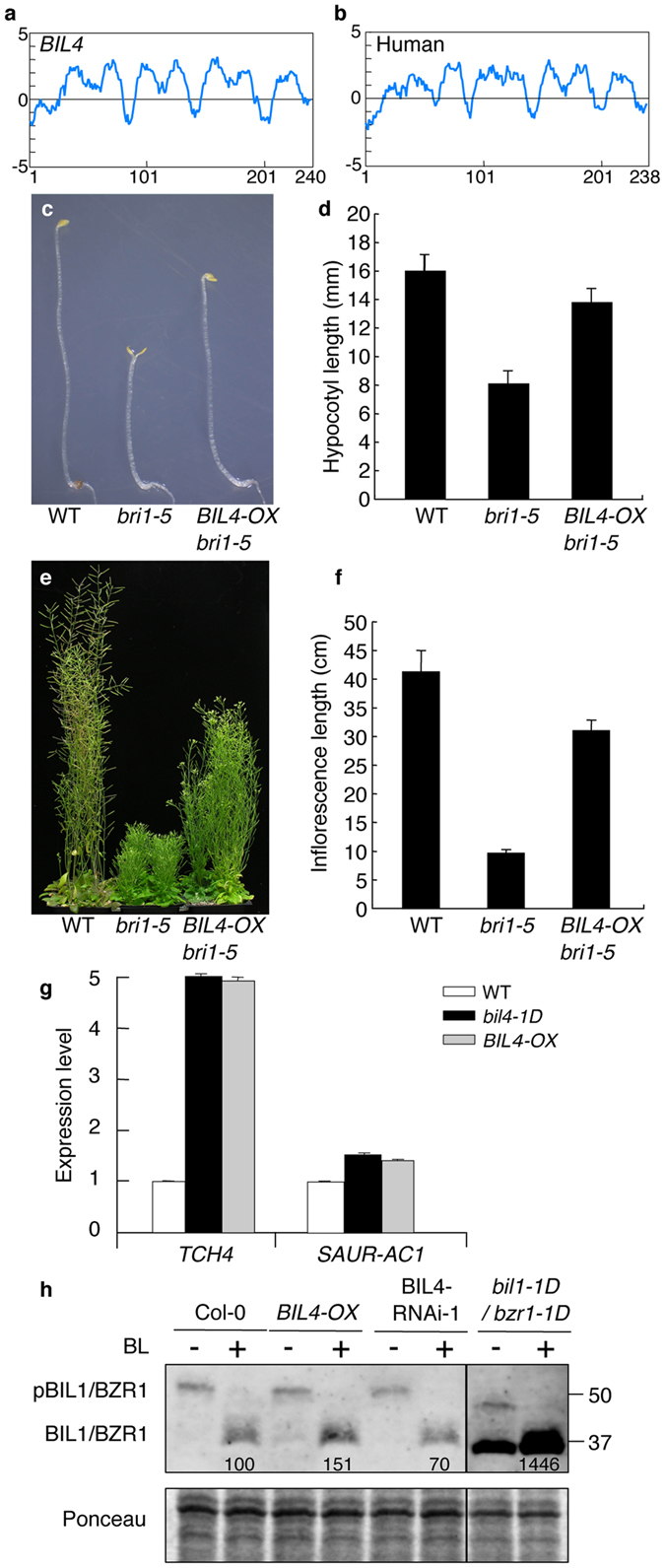
BIL4 is a positive regulator of BR signaling. (**a**,**b**) Hydrophobicity profiles of BIL4 (**a**) and the human homolog Golgi anti-apoptotic protein (hGAAP) (**b**). (**c,d**) Phenotype (**c**) and hypocotyl length (**d**) of wild-type (WT), *bri1-5* and *BIL4-OX bri1-5* double mutant seedlings grown in the dark for 7 days. The results are presented as the mean ± s.d. (n > 30 seedlings). (**e**,**f**) Phenotype (**e**) and inflorescence length (**f**) of WT, *bri1-5* and *BIL4-OX bri1-5* plants grown in soil for 6 weeks. The results are presented as the mean ± s.d. (n > 20 plants). (**g**) qRT-PCR analyses of *TCH4* and *SAUR-AC1* expression levels in wild-type, *bil4-1D*, and *BIL4-OX* plants grown in the dark for 8 days. The results are presented as the mean ± s.d. (**h**) BIL1/BZR1 phosphorylation status in wild-type, *BIL4-OX*, *BIL4-RNAi-1* and *bil1-1D/bzr1-1D* plants (pBIL1/BZR1, phospho-BIL1/BZR1). Plants that were grown on medium containing 3 µM Brz in the dark for 7 days were submerged for 3 hr in medium containing 100 nM BL. Western blot analyses were performed using the anti-BIL1/BZR1 antibody (upper panel). The protein levels were detected using Ponceau S (lower panel). Numbers indicate the relative BIL1/BZR1 signal levels normalized to the Ponceau S-stained protein band.

BIL4 encodes a seven-transmembrane-domain protein with four homologs in *Arabidopsis* (Supplementary Fig. S2a) and homologs in many other plants^27^, such as rice, tomato, maize, poplar, and moss. G protein-coupled receptors (GPCRs) are also seven-transmembrane-domain proteins, although BIL4 does not have a functional GPCR domain. A detailed BLAST search indicated that BIL4 is a member of a gene family that is evolutionarily conserved in plants and animals, including humans and mice, although this protein family has not been well characterized (Fig. 1a,b; Supplementary Fig. S2b,c; Supplementary Table S1).

We next studied the genetic interaction between a *BIL4-OX* and the BR receptor mutant *bri1-5*, which is a weak allele and displays a semi-dwarf phenotype. We found that *BIL4* overexpression partially rescues the short hypocotyl phenotype of *bri1-5* when germinated in the dark (Fig. 1c,d) and the short inflorescences phenotype of *bri1-5* when grown in the light (Fig. 1e,f). These results further suggest that BIL4 is involved in BR signaling.

To determine the effects of *BIL4* overexpression, we analyzed the expression of BR-responsive genes. Using qRT-PCR, in *bil4-1D* and *BIL4-OX* plants, we detected increased transcript levels of two BR-regulated genes, *TCH4* and *SAUR-AC1* ^29^, which are upregulated during BR-regulated cell elongation (Fig. 1g). In addition, the levels of the BR biosynthesis intermediates typhasterol and castasterone were lower in the *BIL4-OX* plant than in the wild-type plants (Supplementary Table S2). Certain BR biosynthesis genes are regulated by feedback inhibition, and the BR levels are suppressed in the BR signaling-activated mutant *bil1/bzr1* ^21^ and upregulated in the BR receptor-deficient mutant *bri1* ^30^. These results indicate that BIL4 acts as a positive factor in BR signaling.

To investigate the regulatory growth effects caused by *BIL4* overexpression in BR signaling, we used immunoblotting analyses to characterize the phosphorylation status of the BR signaling transcription factor BIL1/BZR1, which has been used as a downstream biochemical marker of BR signaling^31^. Without BR treatment, phosphorylated BIL1/BZR1 was detected in the wild-type plants, and dephosphorylated BIL1/BZR1 was detected by BR treatment. With BR treatment, the amount of dephosphorylated BIL1/BZR1 that was detected in the *BIL4-OX* and *bil1-1D/bzr1-1D* ^21, 22^ plants was higher than that in the BR-treated wild-type plants (Fig. 1h). These results indicate that BIL4 acts as a positive regulator in BR signaling.

### BIL4 regulates cell elongation

**Figure 2:**
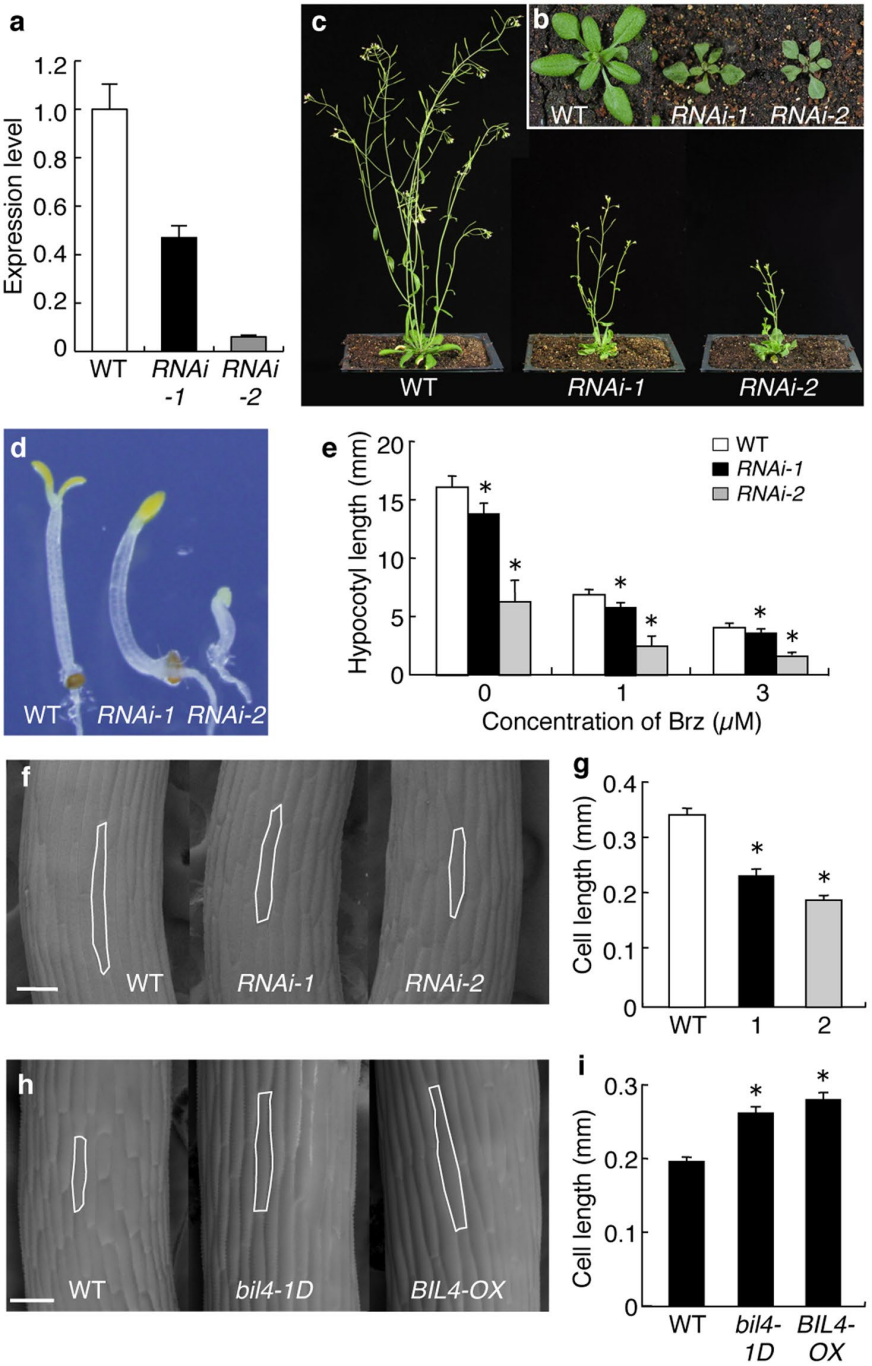
BIL4 is important for cell elongation. (**a**) qRT-PCR analysis of *BIL4* expression in wild-type (WT), *BIL4-RNAi-1* (*RNAi-1*) and *BIL4-RNAi-2* (*RNAi-2*) seedlings grown in the dark for 3 days. The results are presented as the mean ± s.d. (**b**,**c**) Three-week-old (**b**) and 5-week-old (**c**) wild-type and *BIL4-RNAi* plants. (**d**,**e**) Phenotype (**d**) and hypocotyl length (**e**) of wild-type and *BIL4-RNAi* seedlings grown on medium containing 3 µM Brz in the dark for 6 days. The results are presented as the mean ± s.d., n > 30 seedlings. Asterisks indicate a significant difference from the wild-type plant (*P < *0.01 by Student’s *t*-test). (**f** to **i**) SEM images (**f,h**) and length (**g,i**) of the hypocotyl cells. The typical cell phenotype is marked by a white frame. Scale bar, 100 µm. The results are presented as the mean ± s.e.m. Asterisks indicate a significant difference relative to wild-type plants (*P < *0.01 by Student’s *t*-test). *RNAi* seedlings were germinated on 1 µM Brz (n > 17 cells; **f,g**), and overexpressing seedlings were germinated on 3 µM Brz (n > 35 cells; **h** and **i**) in the dark for 7 days.

To investigate the roles of BIL4 in plant development *in vivo*, we examined plants that had been transformed with an RNAi targeting *BIL4* (Fig. 2a). At the adult stage, the *BIL4-RNAi* plants showed shorter inflorescences and smaller leaves than those of the wild-type plants (Fig. 2b,c). The hypocotyls of the *BIL4-RNAi* plants were shorter than those of the wild-type plants grown with and without Brz in the dark (Fig. 2d,e). We also obtained a T-DNA insertion mutant (*BIL4-KO*) (Supplementary Fig. S3a). The T-DNA was inserted approximately 60 bp upstream of the ATG start codon of *BIL4*. The *BIL4-KO* mutant had a phenotype similar to that of wild-type plants grown in control medium, but in the presence of Brz, the *BIL4-KO* mutants had slightly shorter hypocotyls and slightly smaller leaves, thus suggesting that *BIL4-KO* mutants display relatively weak BR-deficient phenotypes (Supplementary Fig. S3b,c). In the *BIL4-RNAi* lines, the expression of *BIL4* homologs *BIL4-H1*, *BIL4-H3*, and *BIL4-H4* was suppressed (Supplementary Fig. S4a). The suppression of *BIL4* and homologous genes in *BIL4-RNAi-2* plants was stronger than that in *BIL4-RNAi-1* plants and was proportional to the strength of the dwarf phenotype in each line. In the *BIL4-RNAi* lines, the expression levels of the BR-upregulated genes *TCH4* and *SAUR-AC1* was decreased (Supplementary Fig. S4b). The BL-stimulated dephosphorylation of BIL1/BZR1 was decreased in *BIL4-RNAi* plants (Fig. 1h). The phenotypes, gene expression patterns, and BIL1/BZR1 phosphorylation statuses of *BIL4-RNAi* plants were similar to those of BR signaling-deficient mutants^11, 32^.

**Figure 3:**
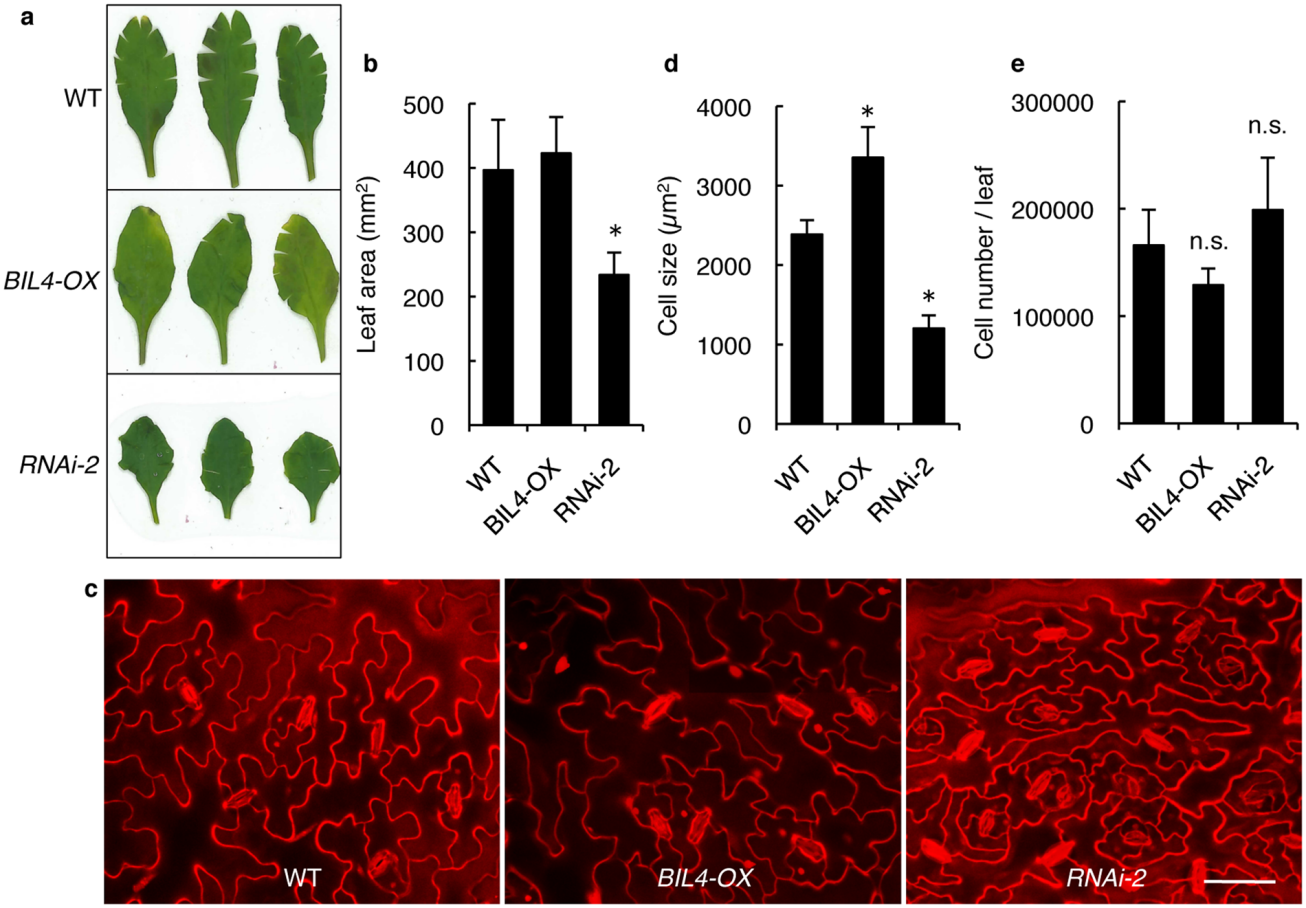
BIL4 positively affects the size of leaf epidermal cells. (**a**) Rosette leaves of wild-type, *BIL4-OX* and *BIL4-RNAi-2* (*RNAi-2*) plants 6 weeks after sowing. (**b**) Leaf area of wild-type, *BIL4-OX* and *RNAi-2* plants. The results are presented as the mean ± s.d., n = 5 leaves. Asterisks indicate a significant difference from the wild-type plants (*P < *0.01 by Student’s *t*-test). (**c**) Confocal images of the epidermis of 6-week-old wild-type, *BIL4-OX* and *RNAi-2* rosette leaves stained with PI. Scale bar, 50 μm. (**d**) Cell size in the epidermis of wild-type, *BIL4-OX* and *RNAi-2* rosette leaves. The results are presented as the mean ± s.d., n = 9 areas. Asterisks indicate a significant difference from the wild-type plant (*P < *0.01 by Student’s *t*-test). (**e**) Epidermal cell number of wild-type, *BIL4-OX* and *RNAi-2* rosette leaves. The results are presented as the mean ± s.d., n = 5 leaves; n.s. indicates no significant difference from the wild-type plants (by Student’s *t*-test).

To investigate whether BIL4 plays a role in plant cell elongation or division *in vivo*, we first performed scanning electron microscopy (SEM) to analyze the hypocotyl cell elongation of *BIL4-OX* and *BIL4-RNAi* plants. The epidermal cells in the hypocotyl regions of the *BIL4-RNAi* plants were shorter than those cells in the wild-type plants when the plants were germinated on 1 μM Brz (Fig. 2f,g). In contrast, *BIL4-OX* exhibited enhanced cell elongation of the epidermal cells in the hypocotyls when the plants were germinated on 3 μM Brz (Fig. 2h,i). These results suggested that BIL4 positively regulates cell elongation in hypocotyls. We next observed the epidermal cell size in the rosette leaves of *BIL4-OX* and *BIL4-RNAi*. The leaf area of *BIL4-RNAi* plants was smaller than that of wild-type plants, and the leaf shape of *BIL4-RNAi* plants was distorted. The size of the leaf area was not different between *BIL4-OX* and wild-type plants (Fig. 3a,b). Nevertheless, the cell area of epidermal cells in the rosette leaf was wider in *BIL4-OX* plants than in wild-type plants. The epidermal cell size in *BIL4-RNAi* was smaller than that in wild-type plants and was proportional to the leaf area (Fig. 3c,d). The cell number in *BIL4-OX* and *BIL4-RNAi* plants was not significantly different from that of wild-type plants (Fig. 3e). These results suggested that BIL4 positively regulates cell elongation on the leaf surface.

### BIL4 is specifically expressed during the activation of cell elongation

**Figure 4:**
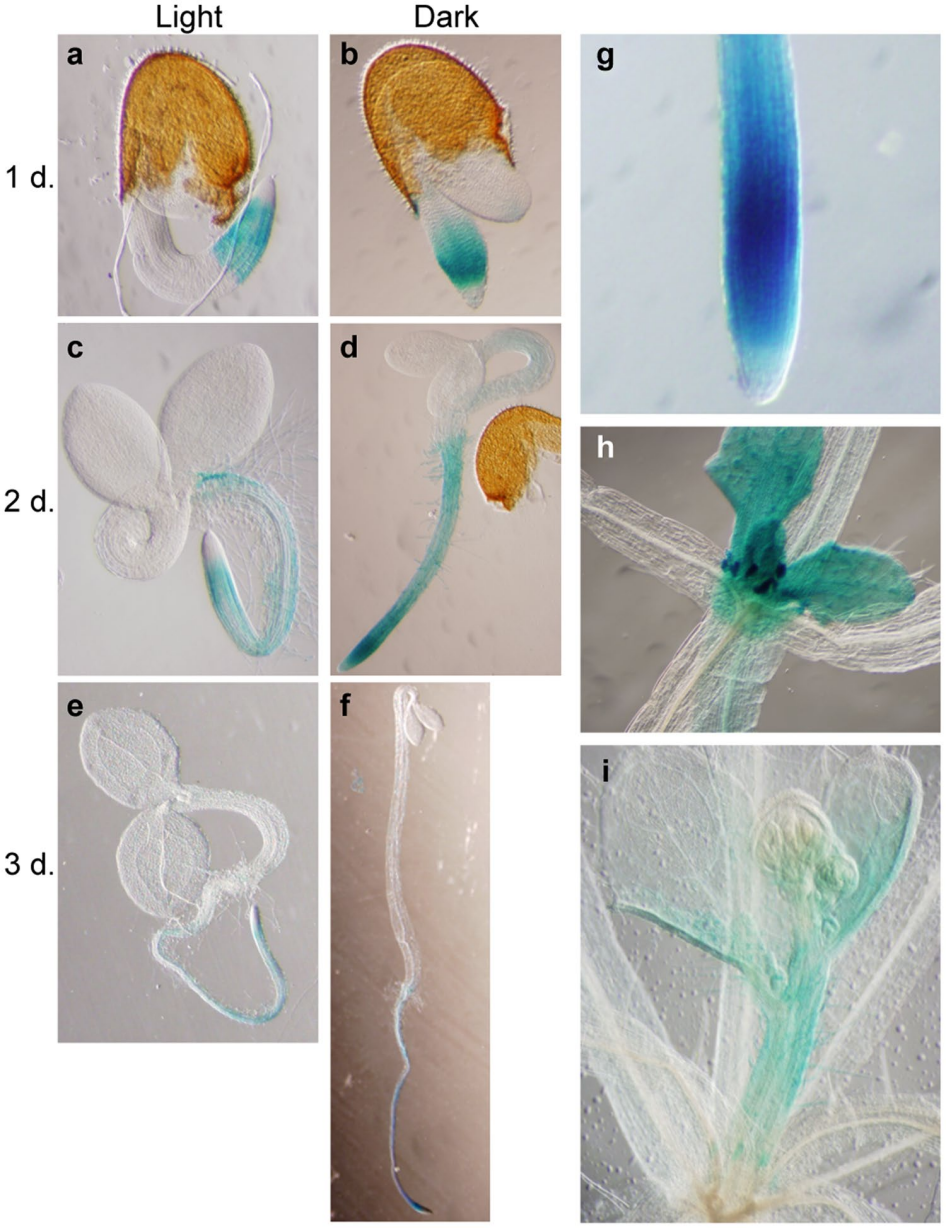
BIL4 is specifically expressed during the activation of cell elongation. (**a**–**f**) *BIL4* promoter (*BIL4pro*)::*GUS* expression pattern 1 (**a**), 2 (**c**) and 3 (**e**) days after germination in the light. *BIL4pro*::*GUS* expression pattern 1 (**b**), 2 (**d**) and 3 (**f**) days after germination in the dark. (**g**–**i**) *BIL4pro*::*GUS* is expressed in the roots (**g**), very small rosette leaves (**h**) and the short bolting stem (**i**).

To gain further insight into the role of BIL4 in plant development and plant cell elongation, we examined *BIL4* expression in each organ and during each growth stage. The *BIL4* promoter region was fused to β-glucuronidase (GUS), and the expression pattern was assessed at different developmental stages. During the germination stage, *BIL4* expression was observed in the juvenile root on day 1 after germination in both the light and dark (Fig. 4a,b). *BIL4* expression in the root was greater near the root apical meristem on day 2 after germination (Fig. 4c,d). *BIL4* expression in the root decreased on day 3 after germination, although the expression near the root apical meristem remained high (Fig. 4e,f). *BIL4* was still highly expressed in the root apical meristem at sites in which both root cell division and expansion occur during the later growth stage (Fig. 4g)^33, 34^. *BIL4* expression was observed in the hypocotyl on day 2 after germination in the dark, a stage in which the cells were actively elongating (Fig. 4d)^35^; in contrast, *BIL4* was not expressed in the short hypocotyls that grew in the light (Fig. 4a,c,e). *BIL4* expression in the hypocotyl was significantly decreased on day 3 after germination in the dark (Fig. 4f). During the adult growth stage, the expression of *BIL4* was detected in young rosette leaves and short inflorescences immediately after the initiation of their development (Fig. 4h,i). These results indicated that *BIL4* is expressed in the very early developmental stages during the activation of cell division and elongation.

### BIL4 localizes to the TGN/EE, LE/MVB, and vacuolar membrane

**Figure 5:**
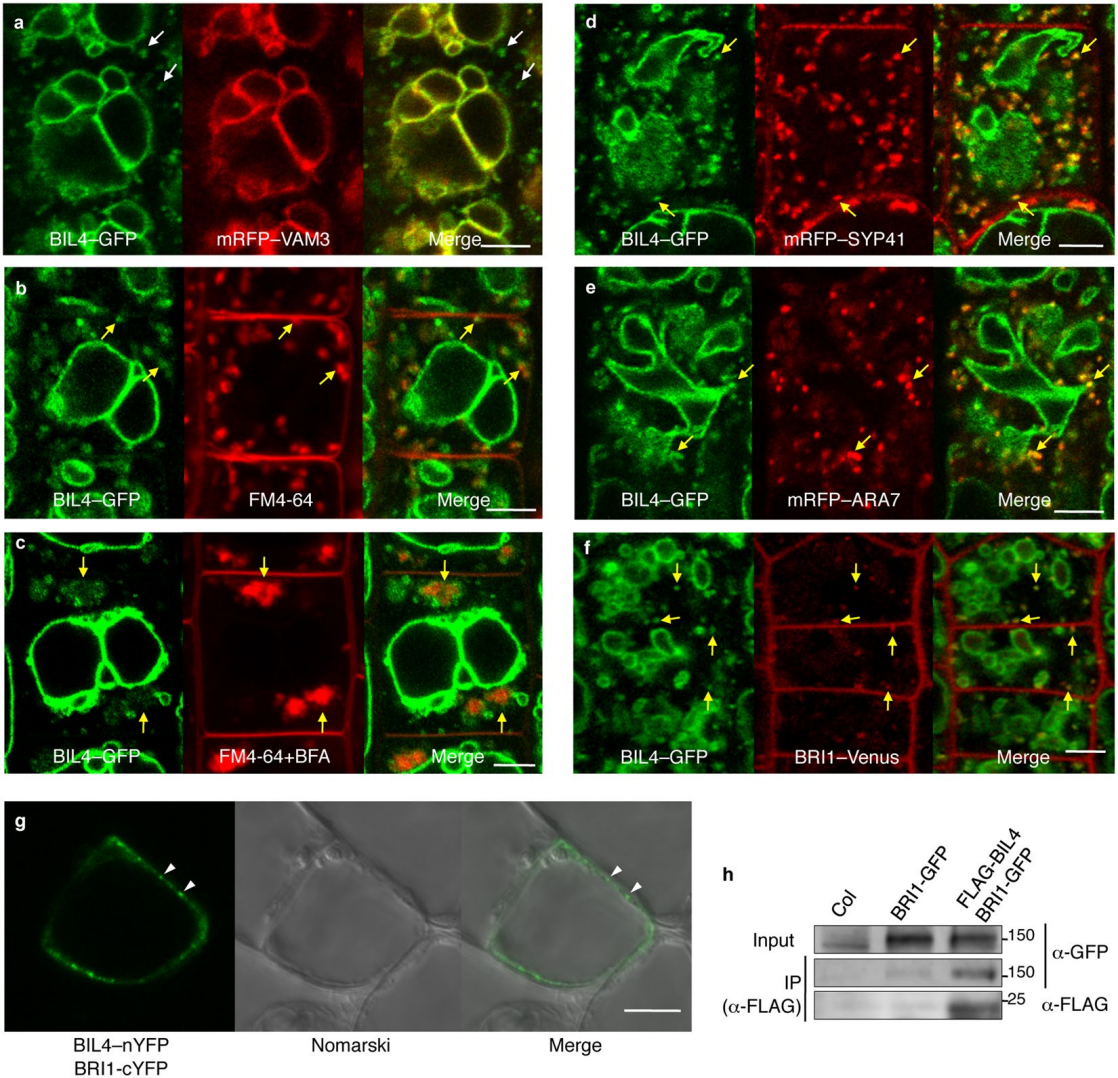
BIL4 is localized to punctate structures and the vacuolar membrane and interacts with BRI1. (**a**) *BIL4*pro::BIL4–GFP partially co-localizes with the vacuolar membrane marker mRFP–VAM3 (unmerged puncta are marked by white arrows). (**b**) *BIL4*pro::BIL4–GFP partially colocalizes with FM4-64. Seedlings were treated with FM4-64 for 5 min and then incubated in water for 40 min. (**c**) Seedlings were pretreated with FM4-64 for 5 min, incubated in water for 20 min and then treated with 50 μM BFA for 20 min. (**d**–**f**) *BIL4*pro::BIL4–GFP partially co-localizes with the TGN/EE marker mRFP–SYP41 (**d**), the LE/MVB marker mRFP–ARA7 (**e**) and *BRI1*pro::BRI1–Venus in the endosome (**f**). Merged structures in b–f are marked by yellow arrows. Scale bar in (**a–f**): 5 µm. (**g**) BiFC assay of the interactions between BIL4 and BRI1 in cultured *Arabidopsis* cells. Scale bars, 10 μm. The white arrowheads indicate characteristic interactions. (**h**) Co-immunoprecipitation of BRI1 and BIL4. Wild-type (Col-0) and transgenic plants coexpressing *BRI1*pro::BRI1–GFP and 35 S::FLAG–BIL4 were grown for 7 days. FLAG–BIL4 was immunoprecipitated by anti-FLAG antibody, and the immunoblots were probed with anti-GFP or anti-FLAG antibody.

To analyze the functions of BIL4 in the cell, we generated transgenic plants harboring a *BIL4* promoter::*BIL4*–*green fluorescent protein* (*GFP*) construct and examined the subcellular localization of BIL4. BIL4–GFP colocalized with the vacuolar membrane marker monomeric red fluorescent protein (mRFP)^36^ VAM3^37^ (Fig. 5a) and γ-tonoplast intrinsic protein (γ-TIP)–mRFP (Supplementary Fig. S5), as indicated by a yellow signal present in the merged image in seedling roots. BIL4–GFP was also associated with mobile and punctate structures that did not colocalize with mRFP–VAM3 of the root cells (Fig. 5a).

The punctate structures visualized with BIL4–GFP colocalized with the endocytic tracer FM4-64 (Fig. 5b). Brefeldin A (BFA) is widely used as a vesicle-trafficking inhibitor^38^ and leads to the aggregation of endosomal compartments. BFA treatment showed that the inhibited trafficking of the BIL4–GFP- and FM4-64-containing vesicles may be BFA dependent (Fig. 5c). BIL4–GFP and the *trans*-Golgi network/early endosome (TGN/EE) marker mRFP–SYP41^39^ were partially colocalized (Fig. 5d). To confirm whether the TGN/EE signal of BIL4–GFP was the result of *de novo* synthesis or the functional role in TGN/EE, we treated the BIL4–GFP and mRFP–SYP41 double transgenic plants with cycloheximide (CHX), which is a protein biosynthesis inhibitor, and/or concanamycin A (ConcA), which is a specific TGN/EE inhibitor acting on vacuolar H^+^-ATPase^40^. Although the endosomal BIL4–GFP signal decreased after CHX treatment, the remaining overlapped BIL4–GFP and mRFP–SYP41 signals after CHX treatment were affected by ConcA (Supplementary Fig. S6a–d). The remaining BIL4–GFP signal after CHX treatment was not from newly synthesized protein.

BIL4–GFP also partially colocalized with the LE/MVB marker mRFP–ARA7 (Fig. 5e). The colocalized signal between BIL4–GFP and mRFP–ARA7 was affected by wortmannin (Wm), which blocks protein cargo trafficking to vacuoles and induces to form ring-like structures of LE/MVB^18^ (Supplementary Fig. S6e). BIL4–GFP co-labeled approximately 58% of the compartments with the TGN/EE marker mRFP–SYP41 and approximately 66% of the compartments with the LE/MVB marker mRFP–ARA7 (Supplementary Fig. S7). BIL4–GFP-labeled punctate structures were often observed adjacent to the Golgi makers *Arabidopsis* β-1,2-xylosyltransferase^41^ (XylT)–mRFP and rat sialyltransferase^42^ (ST)–mRFP (Supplementary Fig. S8). BIL4–GFP was also localized to the punctate signals and vacuolar membrane in the 35 S promoter::BIL4–GFP transformant, similarly to its localization in the BIL4 promoter::BIL4–GFP transformant (Supplementary Fig. S9a–e). The 35 S promoter::BIL4–GFP transgenic plant seedlings showed longer hypocotyls than wild-type seedlings when grown in the dark on medium containing Brz, thus indicating that the fusion protein was functional (Supplementary Fig. S9f). These results suggested that the BIL4 protein localizes to and plays roles in the TGN/EE, LE/MVB, and vacuolar membranes.

### BIL4 interacts with BRI1 in the endosome

In the BR signaling pathway, the BR receptor BRI1 localizes to the plasma membrane and punctate structures^12^. The BRI1–GFP-containing punctate structures were TGN/EE and LE/MVB^43^, which were similar to BIL4–GFP (Fig. 5b–e). To analyze the subcellular localization of BIL4 and BRI1, we generated transgenic plants harboring a *BIL4* promoter::*BIL4*–*GFP* construct and a *BRI1* promoter::*BRI1*–*Venus* construct. BIL4–GFP colocalized with BRI1–Venus in punctate structures (Fig. 5f).

To investigate a possible interaction between BIL4 and BRI1 in plant cells, we introduced plasmids for bimolecular fluorescence complementation (BiFC) into cultured *Arabidopsis* cells. We fused the full-length BIL4 to the N-terminal half of enhanced yellow fluorescence protein (EYFP) (BIL4–nEYFP) and full-length BRI1 to the C-terminal half of EYFP (cEYFP). Both of these constructs were introduced into cultured *Arabidopsis* cells, and the BIL4-BRI1 interaction was monitored by BiFC (Fig. 5g). Because BIL4 and BRI1 were localized in TGN/EE and LE/MVB^43^ (Fig. 5d,e), the detected fluorescence signal may represent TGN/EE and LE/MVB (Fig. 5g). As a negative control, the CERK–cEYFP vector was transformed with the BIL4–nEYFP vector in cultured *Arabidopsis* cells. Although CERK1–cEYFP was expressed, the fluorescence signal of YFP was not detected (Supplementary Fig. S10).

To determine the *in vivo* interaction between BIL4 and BRI1 by using biochemical analyses, we attached full-length BIL4 to the C-terminus of FLAG under the control of the CaMV 35 S promoter and expressed this construct in an *Arabidopsis* BRI1–GFP transformant^44^. The BIL4–FLAG protein was immunoprecipitated by anti-FLAG antibodies in transgenic *Arabidopsis* plants expressing both BRI1–GFP and BIL4–FLAG, and the resulting immunoprecipitates were analyzed by western blotting with anti-FLAG and anti-GFP antibodies. The anti-FLAG antibody immunoprecipitated BIL4–FLAG and coimmunoprecipitated BRI1–GFP, whereas no BRI1–GFP signal was detected using the immunoprecipitates of the transformants expressing BRI1–GFP alone or wild-type *Arabidopsis* (Fig. 5h). These results suggested that the BIL4 protein interacts with the BRI1 protein.

### BIL4 regulates BRI1 trafficking in the plant cell

**Figure 6:**
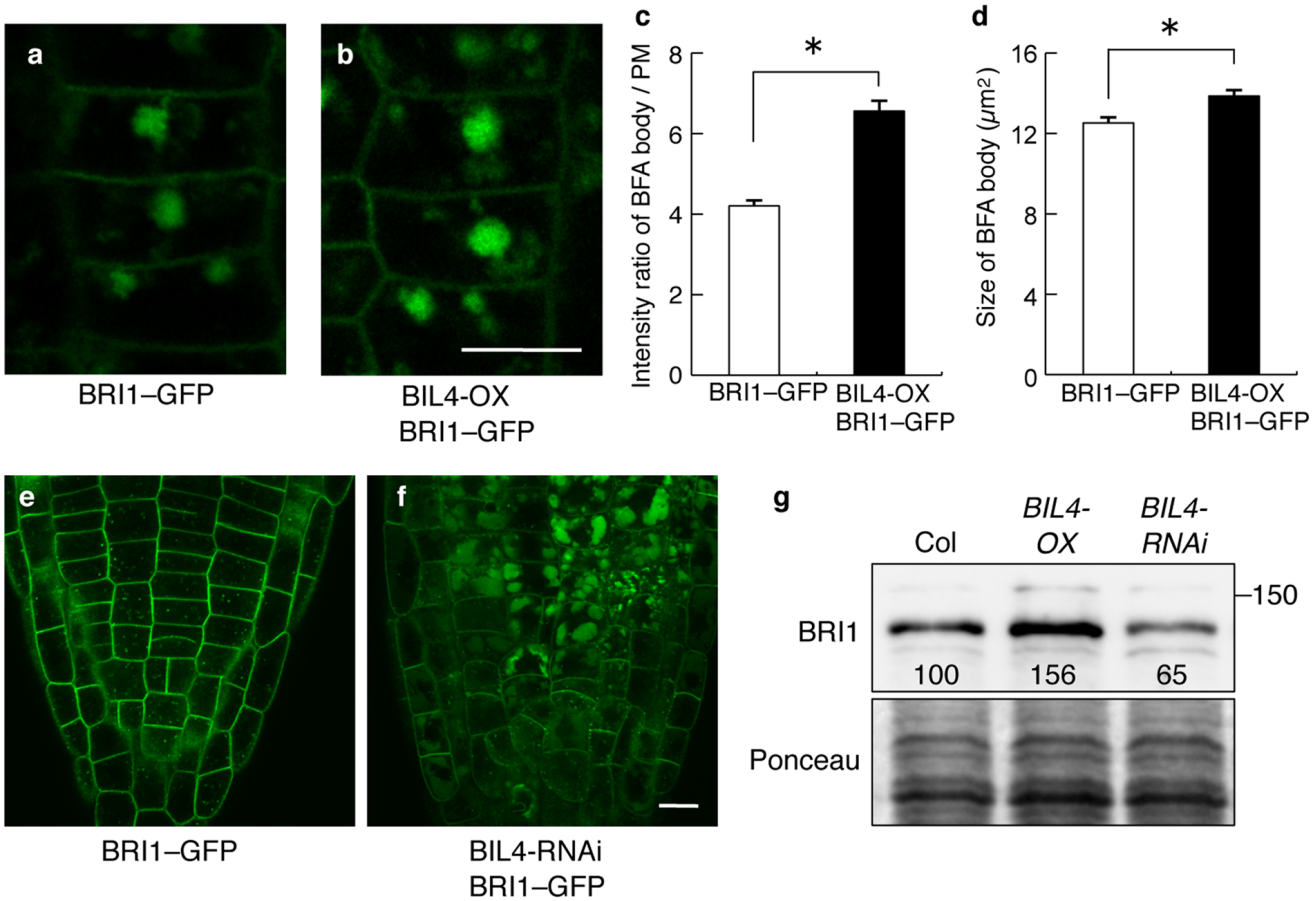
BRI1 subcellular localization is affected in the *BIL4-OX* and *BIL4-RNAi* plants. (**a–d**) Three-day-old seedlings were treated with BFA (50 μM, 0.5 hr). BRI1–GFP-labeled BFA bodies in the wild-type (**a**) and BIL4-OX plants (**b**). Scale bar, 10 µm. Signal intensities of BRI1–GFP-labeled BFA bodies in the wild-type and *BIL4*-*OX* plants (**c**). Sizes of BRI1–GFP-labeled BFA bodies in the wild-type and *BIL4-OX* plants (**d**). (**c,d**) n = 3 roots with at least 30 BFA bodies. *P < 0.01, Student’s *t*-test. Mean ± s.e. (**e**,**f**) BRI1–GFP localization in the root tip of wild-type (**e**) and *BIL4-RNAi* mutant (**f**) 2 days after germination in the dark. Scale bar, 10 µm. (**g**) Plants were grown in the dark for 7 days on medium containing 3 µM Brz. Western blot analyses were performed using the anti-BRI1 antibody (upper panel). The protein levels were detected using Ponceau S (lower panel). Numbers indicate the relative BRI1 signal levels normalized to the Ponceau S-stained protein band.

Recent reports have suggested that BRI1 activates BR signaling when BRI1 is localized to plasma membranes; however, BRI1 undergoes endocytosis to attenuate signaling, thus suggesting that the subcellular localization of BRI1 is associated with the regulation of BR signaling^14^. BRI1 can be internalized and transported to TGN/EE and LE/MVB that are regulated by GNOM^14, 45^. AP-2^15^ and TPLATE^16^ regulate the endocytosis of BRI1 through CME at the plasma membrane. BRI1 is similarly considered to translocate to the vacuoles through processes that are regulated by ubiquitination^17^ and the ESCRT protein^18^, which mediates cargo protein transport to ILVs of LE/MVB. Given that BIL4 interacts with BRI1 in the TGN/EE and/or LE/MVB, BIL4 is predicted to affect the trafficking of BRI1. We subsequently compared the localization of the BRI1–GFP fusion protein in the wild-type, *BIL4-OX*, and *BIL4-RNAi* plants. *BIL4-OX* background increased the number of BRI1–GFP-labeled endosome in epidermal cells of *Arabidopsis* roots (Supplementary Fig. S11). BFA caused the aggregation of BRI1–GFP from the TGN/EE into BFA bodies. The signal intensity and size of these BFA bodies increased in the *BIL4-OX* plants as compared to the wild-type plant background (Fig. 6a–d). In the wild-type plants, BRI1–GFP localized to endosomes and plasma membranes in the root tips (Fig. 6e). In the *BIL4-RNAi* plants, BRI1–GFP strongly localized to the vacuolar lumen in the root tips (Fig. 6f). In an immunoblot analysis, we observed increased BRI1 protein levels in *BIL4-OX* plants compared with those in wild-type plants. In contrast, BRI1 protein levels were decreased in *BIL4-RNAi* plants compared with those in wild-type plants (Fig. 6g). These results suggested that BIL4 inhibits BRI1 trafficking to the vacuoles from the TGN/EE and/or LE/MVB.

## Discussion

Because the BR receptor BRI1 is important in plant development, the detailed mechanism of BRI1-mediated signaling was investigated. BR binds to the extracellular ligand-binding domain of BRI1, which localizes to the plasma membrane^46^, thereby leading to the ligand-dependent dimerization and phosphorylation of BRI1^47^. BRI1 activation induces the dissociation of its inhibitor BKI1 from the plasma membrane and the phosphorylation of BSK1, a positive regulator of the pathway^10, 11^. The BR signals are then transduced to the cytosolic positive phosphatase BSU1^48^ and the negative kinase BIN2^31^, thus leading to the activation of the transcription factors BIL1/BRZ1 and BES1, which regulate more than one thousand different target genes^25, 26^. These results suggest that BRI1 plays important roles in the initiation of BR signaling.

BRI1 localizes not only to plasma membranes but also to endosomes^12^. The inhibition of the ARF-GEF GNOM decreases the endocytosis of BRI1^14^. The knockdown of *Arabidopsis* AP-2 or TPLATE-MUNISCIN-LIKE (TML) protein, a member of the TPC, inhibits the endocytosis of BRI1^15, 16^. The inhibition of GNOM and AP-2 inhibits BRI1 endocytosis, which enhances its localization at the plasma membrane thereby promotes BR signaling^14, 15^. Furthermore, BR treatment does not alter the endocytosis of BRI1^43^. These results suggest that the endocytosis of BRI1 does not promote BR signaling and that the maintenance of BRI1 at the plasma membranes without proceeding to endocytosis or quickly returning BRI1 to the plasma membranes after endocytosis is important for the promotion of BR signaling by BRI1.

In our recent study using a chemical biology approach with Brz^27^, we have identified BIL4, a seven-transmembrane-domain protein, as a positive regulator of BR signaling. BIL4 overexpression in the *bil4-1D* mutant and *BIL4*-*OX* transformant activates the transacting factor BIL1/BZR1 and increases the expression of the BR-induced genes *TCH4* and *SAUR-AC1*. Similarly to the BR receptor BRI1, BIL4 localizes to the TGN/EE and LE/MVB. Our co-immunoprecipitation and BiFC analyses showed that BIL4 interacts with BRI1 in the TGN/EE and LE/MVB.

In *BIL4*-*RNAi* transformants, the vacuole lumen signals of BRI1–GFP, which were difficult to detect in wild-type plants under light conditions, were increased. In *BIL4-OX* transformants, the endosomal localization of BRI1–GFP visualized by BFA treatment increased relative to that of wild-type plants. The translocation of BRI1 to the vacuoles was prevented by *BIL4* overexpression, as indicated by the extensive accumulation of BRI1 in the TGN/EE and LE/MVB and the increase in BFA bodies. These results suggested that BIL4 inhibits the translocation of BRI1 from the TGN/EE to the vacuoles. BIL4 might play important roles for BRI1 trafficking from the TGN/EE and LE/MVB to the vacuoles (Supplementary Fig. S12). The vacuolar transport of BRI1 is considered to be regulated by ubiquitination^17^ and the ESCRT protein^18^. It will be interesting to examine the relationship between these factors and BIL4 in future studies.

*BIL4* is expressed very early in young elongating cells. The overexpression of *BIL4* in the *bil4-1D* mutants and the *BIL4-OX* transformants promotes cell elongation, whereas the deficiency of *BIL4* in the *BIL4-RNAi* transformants inhibits cell elongation. Although the expression of *BIL4* in the hypocotyl was limited to 2 days after germination in the dark, its effect on cell elongation persisted until 7 days after germination. These results were similar to those on the nuclear import of BIL1/BZR1 from the cytosol in the hypocotyl for 96 hr after germination, whose effects persisted until 7 days after germination^21^.

In previous studies, loss-of-function mutants in BR signaling and BR biosynthesis have been reported to exhibit decreased cell size and number of leaf epidermal cells and mesophyll cells. Transformants overexpressing the BR receptor BRI1 show an increased size of leaf epidermal and mesophyll cells^49^. Here, we found that the leaf epidermal cell size increased in *BIL4-OX* plants and decreased in *BIL4-RNAi* plants compared with wild-type plants. These results suggested that BIL4 plays important roles in cell elongation control in BR signaling.

BIL4 is evolutionarily conserved in eukaryotes including plants and animals. The most related protein in humans is the Golgi antiapoptotic protein (hGAAP), which shares 49% amino acid similarity and 27% identity with *Arabidopsis* BIL4. Human hGAAP exhibits 73% identity with the camelpox virus protein vGAAP. hGAAP localizes in the Golgi and is involved in cell death regulation via the modulation of intracellular Ca^2+^ flux^50, 51^. An increase in intracellular calcium is known to trigger vesicle fusion that system relates to SNARE, calmodulin and so on^52–54^. It will be interesting to see if BIL4 could control calcium flux thereby contributing to BRI1 intracellular trafficking.

## Methods

### Plant materials and growth conditions

*Arabidopsis thaliana* ecotype Columbia (Col-0) was used as the wild-type plant. The *bri1-5* mutant was in the *Arabidopsis thaliana* WS ecotype background. *BIL4-KO* mutant plants (CS378201) were obtained from the ABRC. The methods for seed sterilization and the conditions for plant growth have been previously described^27^.

### Quantitative real-time PCR

The methods for total RNA isolation, cDNA synthesis, and real-time PCR have been previously described^27^. The sequences of the gene-specific primers for real-time PCR were as follows: for *TCH4*, 5′-CGAGTCTTTGGAACGCTGAT-3′ and 5′-CTTCTTGTTGAAAGCCACGG-3′; for *SAUR-AC1*, 5′-GAGATATGTGGTGCCGGTTT-3′ and 5′-GTATTGTTAAGCCGCCCATT-3′; for *BIL4*, 5′-CACTCTCTTAAGGGCTGTTGATA-3′ and 5′-TGAACTAGATAACAATGGCACAGT-3′; for *BIL4-H1*, 5′-TGATTCTTGAGGCAGCGATT-3′ and 5′-GGCAAAGACCATGAGAACGA-3′; for *BIL4-H3*, 5′-ACGAGCTGTATCCGGGAATG-3′ and 5′-GGAGACTCCGACGGTGACAA-3′; for *BIL4-H4*, 5′-TTAGCCATGGACAAACCGTA-3′ and 5′-GAGCTGATTCTCGCCGTAA-3′; and for *UBQ2*, 5′-CCAAGATCCAGGACAAAGAAGGA-3′ and 5′-TGGAGACGAGCATAACACTTGC-3′.

### BR measurements

The plants were grown under long-day conditions (16 hr light/8 hr dark) for 30 days. The plants (20 g fresh weight) were extracted twice with 200 ml of MeOH, and deuterium-labeled internal standards (1 ng/g fresh weight) were added to the extract. BR purification and quantification were performed as previously described^55^.

### Transgenic plant generation

The *BIL4-OX*, ST–mRFP, mRFP–VAM3 and mRFP–SYP41 transgenic plants were generated as previously described^27, 37, 56^. To knock down *BIL4* by RNAi, *BIL4* cDNA was amplified from Col-0 cDNA and cloned into the binary vector pGWB80 using a Gateway strategy (Invitrogen, Carlsbad, CA, USA). To observe BIL4 sub-cellular localization under the *BIL4* promoter, a 1920-bp genomic fragment from the *BIL4* promoter region of the coding region of *BIL4* was amplified from Col-0 genomic DNA and cloned using Gateway technology into the binary vector pGWB4^57^, which contains the GFP protein-coding sequence but no promoter. To observe BIL4 sub-cellular localization, the coding sequence of *BIL4* without the stop codon was amplified from Col-0 cDNA and cloned using Gateway technology into the binary vector pGWB5, which contains a 35S promoter and the GFP protein-coding sequence. For the observation of *BIL4* expression in plant organs, a 1460-bp genomic fragment from the *BIL4* promoter region was amplified from Col-0 genomic DNA and cloned using Gateway technology into the binary vector pGWB3^57^ containing the *GUS* coding sequence. The resulting constructs, p*BIL4*–GFP, p*BIL4pro*::*GUS* and p*BIL4*-RNAi, were transformed into Col-0 using the floral dip method. The XylT–mRFP construct was generated by modifying XylT–GFP^41^ and then cloned into pGWB2 (Shoda *et al*., unpublished data). The mRFP–ARA7^58^ construct was cloned into a pBGW vector containing nos-promoter-driven BASTA (glucosinolate) resistance. The plant organelle marker–mRFP constructs were transformed into the *BIL4*–GFP transformants by using the floral dip method.

### GUS staining

The plants expressing the *BIL4* promoter::*GUS* reporter gene fusion were histochemically stained according to the method of Ito and Fukuda, with minor modifications^59^. Digital images were captured using a Leica MZ FLIII stereomicroscope (Leica, Wetzlar, Germany).

### Scanning electron microscopy

The samples were transferred to a low-vacuum scanning electron microscope, and the analysis was performed using a JSM-5600LV microscope (JEOL, Tokyo, Japan).

### Statistical analysis of the root apical meristem

The meristem size in *Arabidopsis* roots was measured as previously described^5^.

### Statistical analysis of the leaf area and epidermal cell size

The largest leaf per plant was measured for 5 plants of each line. Leaves were scanned, and the leaf area was measured with ImageJ software (http://rsb.info.nih.gov/ij/). Leaf regions at 75% of the leaf blade length were stained in 45 μg/ml PI for 30 min, rinsed and mounted in dH_2_O, and cells at the abaxial epidermis were imaged using an LSM700 laser-scanning microscope (Zeiss). One or 2 areas per leaf were measured for 5 of the analyzed plants. The number of cells (150 cells on average) per drawn image area was counted in ImageJ. The average cell size was calculated from the ratio of the image area/cell number. The total cell number in the leaves was calculated from the multiplication of cell number per mm^2^ by total leaf area.

### Inhibitor treatments and FM4-64 staining

FM4-64 (Molecular Probes, Carlsbad, CA, USA) was dissolved in dimethyl sulfoxide (DMSO) and applied to *Arabidopsis* plants at a final concentration of 4 µM for 3 min. The plants were washed with water to remove the excess dye and subsequently examined. BFA (Sigma-Aldrich, St. Louis, MO) was dissolved in DMSO, and the solution was applied to plants at a final concentration of 50 µM for 30 min. Four-day-old seedlings were incubated in distilled water containing 2 µM ConcA, 50 µM CHX, or 33 µM Wm. The following stock solutions were used: 1 mM ConcA in DMSO, 50 mM CHX in DMSO, and 33 mM Wm in DMSO.

### Confocal laser scanning microscopy

The stable double transformants were observed using a Zeiss LSM700 or LSM780 confocal laser-scanning microscope (Zeiss, Jena, Germany).

### Transformation of the *Arabidopsis* suspension culture line

An *Arabidopsis* suspension culture was transformed as previously described^60^ with minor modifications. Transformed *Agrobacterium* that was cultured in YEP medium containing appropriate antibiotics was suspended in modified MS medium containing acetosyringone. The *Agrobacterium* suspensions were inoculated into 10 ml of 2-day-old cultured Alex cells of *Arabidopsis*. To remove the *Agrobacterium*, 50 µl of 100 mg/ml claforan was added to these cultures 3 days after inoculation. Microscopy was performed 4 days after *Agrobacterium* inoculation. To construct the BiFC vector, the BIL4 coding region without stop codon was cloned into the Gateway binary vector pB4GWnY (from Dr. T. Mano, National Institute for Basic Biology) to generate BIL4-nEYFP, and the BRI1 and CERK1 coding regions without a stop codon were cloned into the Gateway binary vector pB4GWcY (from Dr. T. Mano, National Institute for Basic Biology) to generate BRI1-cEYFP and CERK1-cEYFP.

### Co-immunoprecipitation

The proteins were extracted by grinding 5 g of 7-day-old seedlings in liquid N_2_, and further grinding in 14 ml of cold 50 mM Tris-HCl, pH 7.5, 100 mM NaCl, 2 mM EDTA, 1% CHAPS, and protease inhibitor cocktail tablets (Roche Diagnostics). The extract was centrifuged twice at 10,000 x *g* for 5 min (4 °C), and the resulting supernatant was cleared by filtration through a 0.8-µm Millex-AA filter (Millipore, Billerica, MA). The proteins in the supernatant were quantified using the Bio-Rad Protein Assay reagent and were adjusted to the same concentration prior to immunoprecipitation. Flag–BIL4 was immunoprecipitated with prewashed anti-Flag M2 affinity gel (Sigma-Aldrich) at 4 °C for 30 min, washed extensively, and eluted with 70 µl of 2 × LDS sample loading buffer (3% LDS, 60 mM Tris-HCl, pH 6.8, 6% [w/v] sucrose, and 0.003% [w/v] BPB). After incubation at 25 °C for 30 min, the proteins were resolved by SDS-PAGE. The immunoprecipitated proteins were detected by immunoblot analysis on Hybond ECL nitrocellulose membranes (GE Healthcare) using a monoclonal anti-Flag M2 antibody (Sigma-Aldrich) at a 1:1000 dilution and an anti-GFP antibody (Molecular Probes) at a 1:1200 dilution. The blots were developed using horseradish peroxidase (HRP)-linked secondary antibodies and the Immobilon Western Chemiluminescent HRP substrate (Millipore).

### Immunoblot analysis

Proteins were extracted by grinding 100 mg of plants in liquid N_2_, and further grinding in 1x SDS buffer. The proteins were detected by immunoblot analysis on Hybond ECL nitrocellulose membranes (GE Healthcare) with different antibodies using Western Blot Immuno Booster Solution (Takara). The antibodies used for immunodetections were anti-BIL1/BZR1 (1:5000) and anti-BRI1 (1:5000, Agrisera). The secondary antibody was anti-rabbit-HRP (1:15000, Promega).

### Imaging and image analysis of BFA bodies

The imaging zone was maintained to be consistent with that showing the epidermal cells of the root tip meristematic zone, 10–15 cells above the quiescent center. To measure the fluorescence signal in BFA bodies, we obtained 2–4 slices of epidermal cells. Image analysis and signal quantification were performed using the measurement function of the LSM software ZEN 2009 (Zeiss). The signal intensity ratio of a BFA-body region was quantified, normalized to the area, and divided by the signal intensity of a nearby plasma membrane.

## Electronic supplementary material

Supplemental Figure & Table

## References

[1] Norman AW, Mizwicki MT, Norman DP (2004). Steroid-hormone rapid actions, membrane receptors and a conformational ensemble model. Nat Rev Drug Discov.

[2] Azpiroz R, Wu Y, LoCascio JC, Feldmann KA (1998). An Arabidopsis brassinosteroid-dependent mutant is blocked in cell elongation. Plant Cell.

[3] Nakashita H (2003). Brassinosteroid functions in a broad range of disease resistance in tobacco and rice. Plant J.

[4] Yamamoto R (2007). Co-regulation of brassinosteroid biosynthesis-related genes during xylem cell differentiation. Plant Cell Physiol.

[5] Gonzalez-Garcia MP (2011). Brassinosteroids control meristem size by promoting cell cycle progression in Arabidopsis roots. Development.

[6] Bekh-Ochir D (2013). A novel mitochondrial DnaJ/Hsp40 family protein BIL2 promotes plant growth and resistance against environmental stress in brassinosteroid signaling. Planta.

[7] Clouse SD, Langford M, McMorris TC (1996). A brassinosteroid-insensitive mutant in Arabidopsis thaliana exhibits multiple defects in growth and development. Plant Physiol.

[8] Li J (2002). BAK1, an Arabidopsis LRR receptor-like protein kinase, interacts with BRI1 and modulates brassinosteroid signaling. Cell.

[9] Nam KH, Li J (2002). BRI1/BAK1, a receptor kinase pair mediating brassinosteroid signaling. Cell.

[10] Tang W (2008). BSKs mediate signal transduction from the receptor kinase BRI1 in Arabidopsis. Science.

[11] Wang X, Chory J (2006). Brassinosteroids regulate dissociation of BKI1, a negative regulator of BRI1 signaling, from the plasma membrane. Science.

[12] Russinova E (2004). Heterodimerization and endocytosis of Arabidopsis brassinosteroid receptors BRI1 and AtSERK3 (BAK1). Plant Cell.

[13] Platta HW, Stenmark H (2011). Endocytosis and signaling. Curr Opin Cell Biol.

[14] Irani NG (2012). Fluorescent castasterone reveals BRI1 signaling from the plasma membrane. Nat Chem Biol.

[15] Di Rubbo S (2013). The clathrin adaptor complex AP-2 mediates endocytosis of brassinosteroid insensitive1 in Arabidopsis. Plant Cell.

[16] Gadeyne A (2014). The TPLATE adaptor complex drives clathrin-mediated endocytosis in plants. Cell.

[17] Martins S (2015). Internalization and vacuolar targeting of the brassinosteroid hormone receptor BRI1 are regulated by ubiquitination. Nat Commun.

[18] Cardona-Lopez X (2015). ESCRT-III-Associated Protein ALIX Mediates High-Affinity Phosphate Transporter Trafficking to Maintain Phosphate Homeostasis in Arabidopsis. Plant Cell.

[19] Asami T, Yoshida S (1999). Brassinosteroid biosynthesis inhibitors. Trends Plant Sci.

[20] Asami T (2001). Selective interaction of triazole derivatives with DWF4, a cytochrome P450 monooxygenase of the brassinosteroid biosynthetic pathway, correlates with brassinosteroid deficiency in planta. J Biol Chem.

[21] Wang ZY (2002). Nuclear-localized BZR1 mediates brassinosteroid-induced growth and feedback suppression of brassinosteroid biosynthesis. Dev Cell.

[22] Asami T, Nakano T, Fujioka S (2005). Plant brassinosteroid hormones. Vitam Horm.

[23] Yin Y (2002). BES1 accumulates in the nucleus in response to brassinosteroids to regulate gene expression and promote stem elongation. Cell.

[24] He JX (2005). BZR1 is a transcriptional repressor with dual roles in brassinosteroid homeostasis and growth responses. Science.

[25] Yin Y (2005). A new class of transcription factors mediates brassinosteroid-regulated gene expression in Arabidopsis. Cell.

[26] Sun Y (2010). Integration of brassinosteroid signal transduction with the transcription network for plant growth regulation in Arabidopsis. Dev Cell.

[27] Yamagami A (2009). Chemical genetics reveal the novel transmembrane protein BIL4, which mediates plant cell elongation in brassinosteroid signaling. Biosci Biotechnol Biochem.

[28] Asami T (2000). Characterization of brassinazole, a triazole-type brassinosteroid biosynthesis inhibitor. Plant Physiol.

[29] Goda H, Shimada Y, Asami T, Fujioka S, Yoshida S (2002). Microarray analysis of brassinosteroid-regulated genes in Arabidopsis. Plant Physiol.

[30] Noguchi T (1999). Brassinosteroid-insensitive dwarf mutants of Arabidopsis accumulate brassinosteroids. Plant Physiol.

[31] He JX, Gendron JM, Yang Y, Li J, Wang ZY (2002). The GSK3-like kinase BIN2 phosphorylates and destabilizes BZR1, a positive regulator of the brassinosteroid signaling pathway in Arabidopsis. Proc Natl Acad Sci USA.

[32] Yu X, Li L, Guo M, Chory J, Yin Y (2008). Modulation of brassinosteroid-regulated gene expression by Jumonji domain-containing proteins ELF6 and REF6 in Arabidopsis. Proc Natl Acad Sci USA.

[33] Beemster GT, Fiorani F, Inze D (2003). Cell cycle: the key to plant growth control?. Trends Plant Sci.

[34] Birnbaum K (2003). A gene expression map of the Arabidopsis root. Science.

[35] Gendreau E (1997). Cellular basis of hypocotyl growth in Arabidopsis thaliana. Plant Physiol.

[36] Campbell RE (2002). A monomeric red fluorescent protein. Proc Natl Acad Sci USA.

[37] Uemura T (2010). Vacuolar/pre-vacuolar compartment Qa-SNAREs VAM3/SYP22 and PEP12/SYP21 have interchangeable functions in Arabidopsis. Plant J.

[38] Robineau S, Chabre M, Antonny B (2000). Binding site of brefeldin A at the interface between the small G protein ADP-ribosylation factor 1 (ARF1) and the nucleotide-exchange factor Sec7 domain. Proc Natl Acad Sci USA.

[39] Uemura T (2004). Systematic analysis of SNARE molecules in Arabidopsis: dissection of the post-Golgi network in plant cells. Cell Struct Funct.

[40] Dettmer J, Hong-Hermesdorf A, Stierhof YD, Schumacher K (2006). Vacuolar H+-ATPase activity is required for endocytic and secretory trafficking in Arabidopsis. Plant Cell.

[41] Pagny S (2003). Structural requirements for Arabidopsis beta1,2-xylosyltransferase activity and targeting to the Golgi. Plant J.

[42] Boevink P (1998). Stacks on tracks: the plant Golgi apparatus traffics on an actin/ER network. Plant J.

[43] Geldner N, Hyman DL, Wang X, Schumacher K, Chory J (2007). Endosomal signaling of plant steroid receptor kinase BRI1. Genes Dev.

[44] Friedrichsen DM, Joazeiro CA, Li J, Hunter T, Chory J (2000). Brassinosteroid-insensitive-1 is a ubiquitously expressed leucine-rich repeat receptor serine/threonine kinase. Plant Physiol.

[45] Viotti C (2010). Endocytic and secretory traffic in Arabidopsis merge in the trans-Golgi network/early endosome, an independent and highly dynamic organelle. Plant Cell.

[46] Kinoshita T (2005). Binding of brassinosteroids to the extracellular domain of plant receptor kinase BRI1. Nature.

[47] Wang X (2008). Sequential transphosphorylation of the BRI1/BAK1 receptor kinase complex impacts early events in brassinosteroid signaling. Dev Cell.

[48] Kim TW (2009). Brassinosteroid signal transduction from cell-surface receptor kinases to nuclear transcription factors. Nat Cell Biol.

[49] Zhiponova MK (2013). Brassinosteroid production and signaling differentially control cell division and expansion in the leaf. New Phytol.

[50] de Mattia F (2009). Human Golgi antiapoptotic protein modulates intracellular calcium fluxes. Mol Biol Cell.

[51] Gubser C (2007). A new inhibitor of apoptosis from vaccinia virus and eukaryotes. PLoS Pathog.

[52] Peters C, Mayer A (1998). Ca2+/calmodulin signals the completion of docking and triggers a late step of vacuole fusion. Nature.

[53] Piper RC, Luzio JP (2004). CUPpling calcium to lysosomal biogenesis. Trends Cell Biol.

[54] Morgan AJ, Platt FM, Lloyd-Evans E, Galione A (2011). Molecular mechanisms of endolysosomal Ca2+ signalling in health and disease. Biochem J.

[55] Fujioka S, Takatsuto S, Yoshida S (2002). An early C-22 oxidation branch in the brassinosteroid biosynthetic pathway. Plant Physiol.

[56] Uemura T (2012). Qa-SNAREs localized to the trans-Golgi network regulate multiple transport pathways and extracellular disease resistance in plants. Proc Natl Acad Sci USA.

[57] Nakagawa T (2007). Development of series of gateway binary vectors, pGWBs, for realizing efficient construction of fusion genes for plant transformation. J Biosci Bioeng.

[58] Asaoka R (2013). Arabidopsis RABA1 GTPases are involved in transport between the trans-Golgi network and the plasma membrane, and are required for salinity stress tolerance. Plant J.

[59] Ito J, Fukuda H (2002). ZEN1 is a key enzyme in the degradation of nuclear DNA during programmed cell death of tracheary elements. Plant Cell.

[60] Saito C (2011). The occurrence of ‘bulbs’, a complex configuration of the vacuolar membrane, is affected by mutations of vacuolar SNARE and phospholipase in Arabidopsis. Plant J.

