# Supplementary material for: Evolutionarily conserved BIL4 suppresses the degradation of brassinosteroid receptor BRI1 and regulates cell elongation
Source: Sci Rep. 2017 Jul 18;7:5739. doi: 10.1038/s41598-017-06016-2 (PMC5515986; doi:10.1038/s41598-017-06016-2)
Supplement: Supplementary file 1 — Supplemental Figure & Table [file 41598_2017_6016_MOESM1_ESM.pdf]

Title

Evolutionarily conserved BIL4 suppresses the degradation of brassinosteroid receptor BRI1 that regulates cell elongation.

Authors

Ayumi Yamagami[1], Chieko Saito[2,3], Miki Nakazawa[4], Shozo Fujioka[1,7], Tomohiro Uemura[3], Minami Matsui[5], Masaaki Sakuta[6], Kazuo Shinozaki[1], Hiroyuki Osada[7], Akihiko Nakano[2,3,8], Tadao Asami[1,9,10,11] and Takeshi Nakano[1,11,*]

[1]Gene Discovery Research Group, RIKEN Center for Sustainable Resource Science, Wako, Saitama 351-0198, Japan
[2]Molecular Membrane Biology Laboratory, RIKEN, Wako, Saitama 351-0198, Japan
[3]Department of Biological Sciences, Graduate School of Science, The University of Tokyo, Hongo, Bunkyo-ku, Tokyo 113-0033, Japan
[4]RIKEN Genomic Science Center, Tsurumi, Yokohama, Kanagawa 230-0045, Japan
[5]Synthetic Genomics Research Group, RIKEN Center for Sustainable Resource Science, Tsurumi, Yokohama, Kanagawa 230-0045, Japan
[6]Department of Biological Sciences, Ochanomizu University, Ohtsuka, Bunkyo-ku, Tokyo 112-8610, Japan
[7]Chemical Biology Research Group, RIKEN Center for Sustainable Resource Science, Wako, Saitama 351-0198, Japan
[8]Live Cell Super-Resolution Imaging Research Team, RIKEN Center for Advanced Photonics, Wako, Saitama 351-0198, Japan
[9]Department of Applied Biological Chemistry, The University of Tokyo, Yayoi, Bunkyo-ku, Tokyo 113-8657, Japan
[10]King Abdulaziz University, Department of Biochemistry, Jeddah 21589, Saudi Arabia
[11]CREST, JST (Japan Science and Technology Agency), Kawaguchi, Saitama 332-0012, Japan
*Corresponding author. E-mail: tnakano@riken.jp

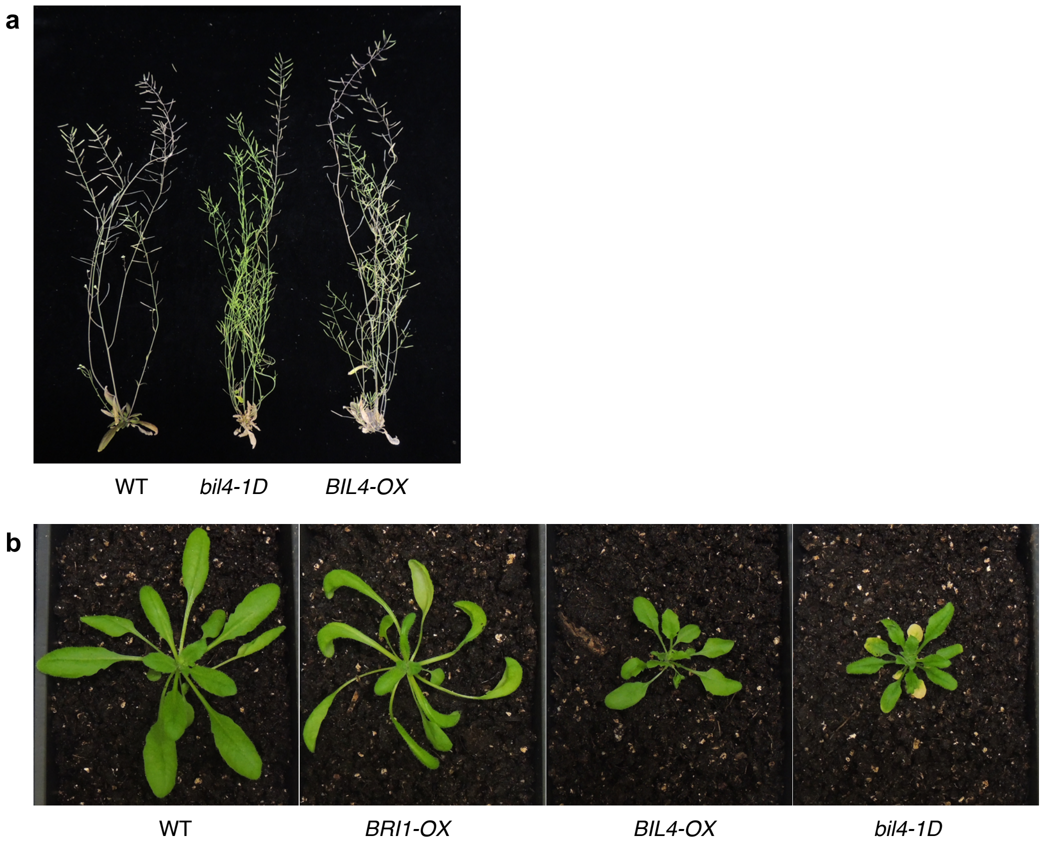

Figure S1 | Phenotype of plants overexpressing *BIL4*. (a) Phenotypes of wild-type (WT), *bil4-1D* and *BIL4-OX* plants grown in soil for 6 weeks. (b) Phenotypes of rosette leaves of WT, *BRI1-OX*, *BIL4-OX, and bil4-1D* plants grown in soil for 30 days.

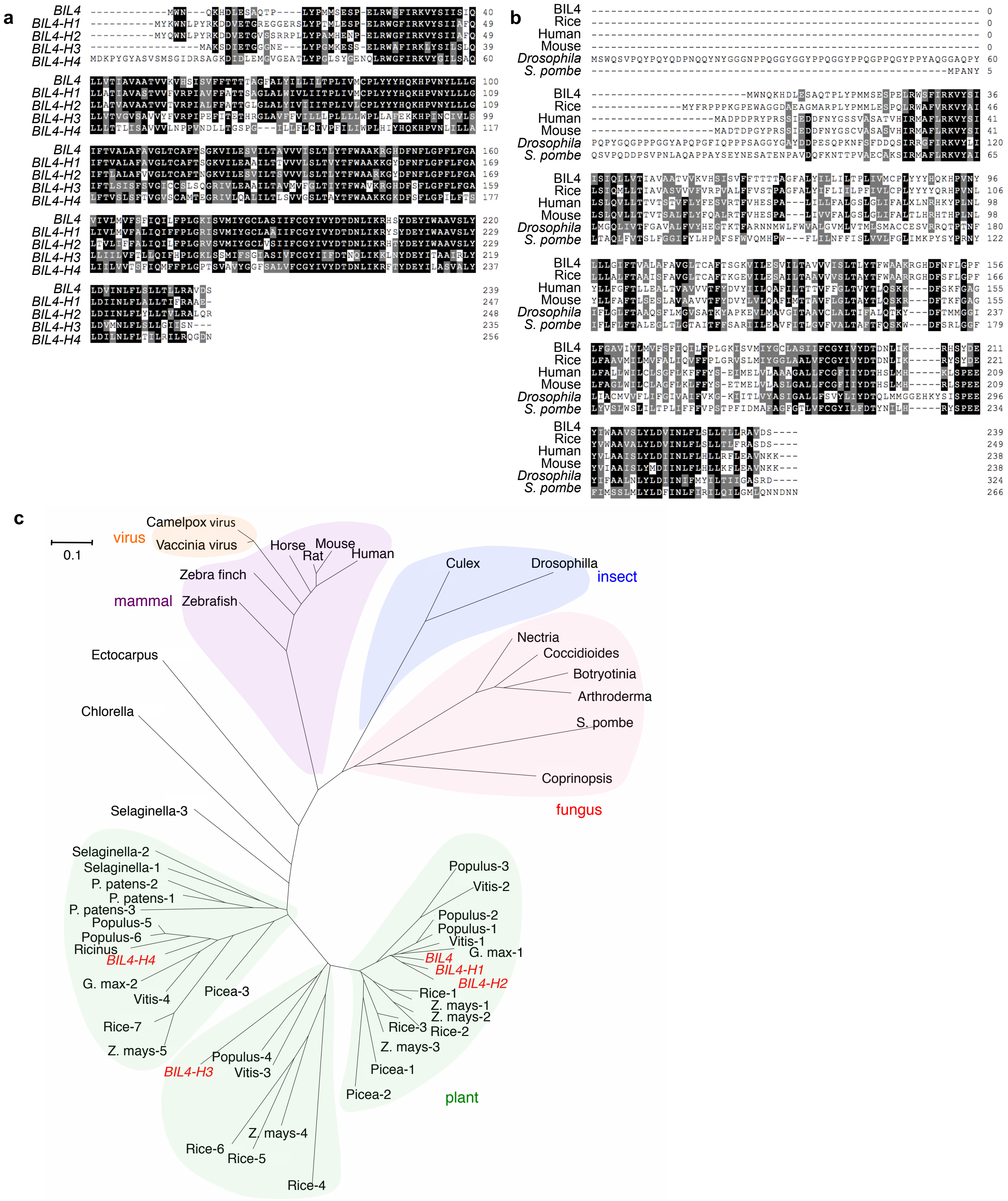

Figure S2 | *Arabidopsis* BIL4 and BIL4 homologs from diverse eukaryotes contain conserved amino acid sequences. (**a**,**b**) Multi-sequence alignment of the homologous BIL4 and BIL4 proteins of *Arabidopsis* (**a**) and of rice, human, mouse, *Drosophila* and *Schizosaccharomyces pombe* (*S. pombe*) (**b**) using ClustalW (v. 1.83). Identical and conserved amino acids are indicated by reversed and shaded characters, respectively. (**c**) A phylogenic tree of *Arabidopsis BIL4* (At3g63310), *BIL4-H1* (At3g03070), *BIL4-H2* (At4g02690), *BIL4-H3* (At4g14730), *BIL4-H4* (At4g15470) and related proteins in other species. (Populus, *Populus trichocarpa*; Vitis, *Vitis vinifera*; *G. max*, *Glycine max*; Rice, *Oryza sativa Japonica Group*; *Z. mays*, *Zea mays*; Picea, *Picea sitchensis*; Ricinus, *Ricinus communis*; *P. patens*, *Physcomitrella patens subsp. patens*; Selaginella, *Selaginella moellendorffii*; Chlorella, *Chlorella variabilis*; Zebrafish, *Danio rerio*; Zebra finch, *Taeniopygia guttata*; Horse, *Equus caballus*; Rattus, *Rattus norvegicus*; Mouse, *Mus musculus*; Human, *Homo sapiens*; Culex, *Culex quinquefasciatus*; Drosophila, *Drosophila melanogaster*; Nectria, *Nectria haematococca mpVI 77-13-4*; Coccidioides, *Coccidioides immitis RS*; Botryotinia, *Botryotinia fuckeliana B05.10*; Arthroderma, *Arthroderma otae CBS 113480*; *S. pombe*, *Schizosaccharomyces pombe 972h-*; Coprinopsis, *Coprinopsis cinerea okayama 7 #130*).

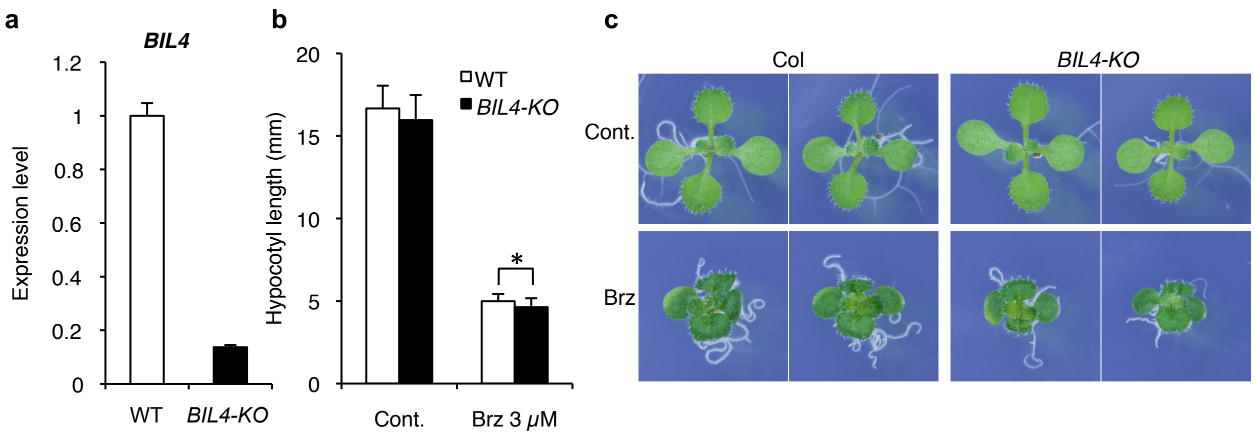

Figure S3 | The *bil4-1* mutant shows weak BR-deficient phenotypes. (**a**) qRT-PCR analysis of *BIL4* expression in wild-type (WT) and *BIL4-KO* grown in soil for 4 weeks. The results are presented as the mean ± s.d. (**b**) Hypocotyl length of WT and *BIL4-KO* seedlings grown on medium containing 3 μM Brz in the dark for 7 days. The results are presented as the mean ± s.d., n = 30 seedlings. Asterisks indicate a significant difference from the wild-type plant ($P < 0.01$ by Student's *t*-test). (**c**) Phenotype of WT and *BIL4-KO* seedlings grown in the light for 10 days on medium containing 3 μM Brz.

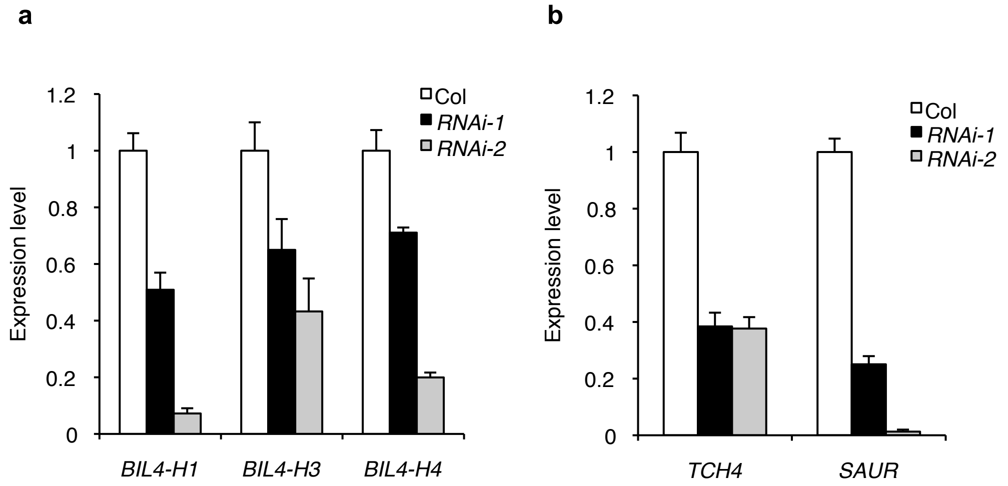

Figure S4 | Expression analysis of BIL4 homologs and BR biosynthesis genes in *BIL4-RNAi* plants. (a,b) Quantitative RT-PCR analyses were performed to compare the *BIL4-H1*, *BIL4-H3*, *BIL4-H4* (a), *TCH4* and *SAUR-AC1* expression levels in wild-type, *BIL4-RNAi-1* (*RNAi-1*), and *BIL4-RNAi-2* (*RNAi-2*) (b) plants grown in the dark for 3 days. The error bars represent the s.d. of four technical repeats.

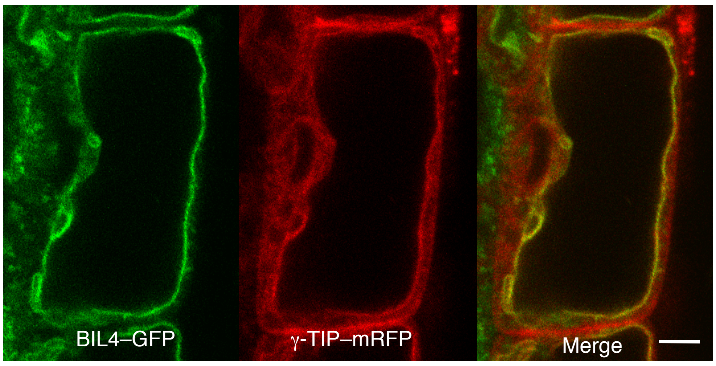

Figure S5 | BIL4 localizes to the vacuolar membrane. BIL4–GFP expressed under the BIL4 promoter is colocalized with the vacuolar membrane marker protein γ-TIP–mRFP. Scale bars, 5 μm.

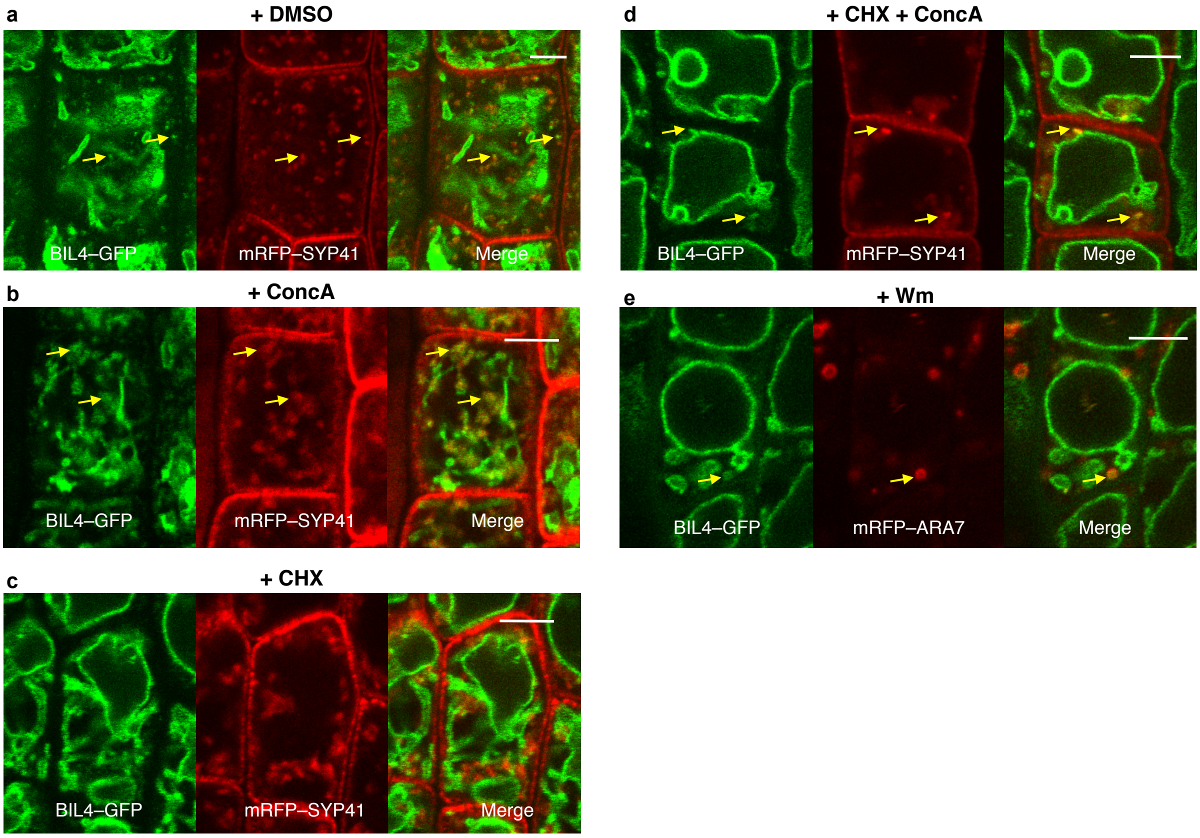

Figure S6 | BIL4 localizes to the TGN/EE and the LE/MVB. (a–d) Drug sensitivity of *BIL4*pro::BIL4–GFP mRFP–SYP41 plants. Four-day-old seedlings were treated with DMSO (a), ConcA (2 µM, 2 hr) (b), CHX (50 µM, 1 hr) (c), and CHX + ConcA (50 µM CHX, 1 hr, and this was followed by treatment with 2 µM ConcA + 50 µM CHX, 1 hr) (d). Arrows show colocalization of BIL4–GFP and mRFP–SYP41. (**e**) Wm sensitivity of *BIL4*pro::BIL4–GFP mRFP–ARA7 plants. Four-day-old seedlings were treated with Wm (33 µM, 2 hr). Arrows show colocalization of BIL4–GFP and mRFP–ARA7. Scale bars, 5 µm.

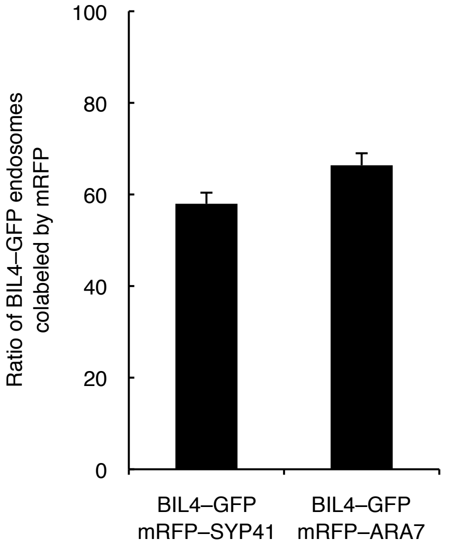

Figure S7 | The relative ratio of BIL4 localization to TGN/EE and the LE/MVB. Ratio of BIL4–GFP endosomes co-labeled with the TGN/EE marker mRFP–SYP41 and the LE/ MVB marker mRFP–ARA7. Endosomes with BIL4–GFP and endomembrane markers were evaluated and counted by ImageJ software in root-tip cells from *BIL4* promoter::*BIL4–GFP mRFP–ARA7* and *BIL4* promoter::*BIL4–GFP mRFP–SYP41* seedlings. The results are presented as the mean ± s.e., n = 20 cells.

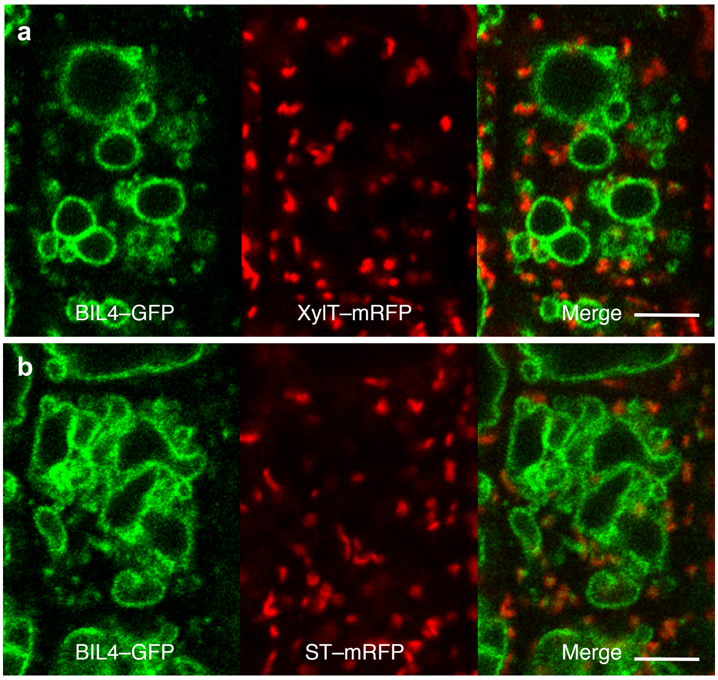

Figure S8 | BIL4–GFP is not localized to the Golgi apparatus. BIL4–GFP expressed under the BIL4 promoter is not colocalized with the Golgi marker XylT–mRFP (**a**) or ST–mRFP (**b**). Scale bars, 5 μm.

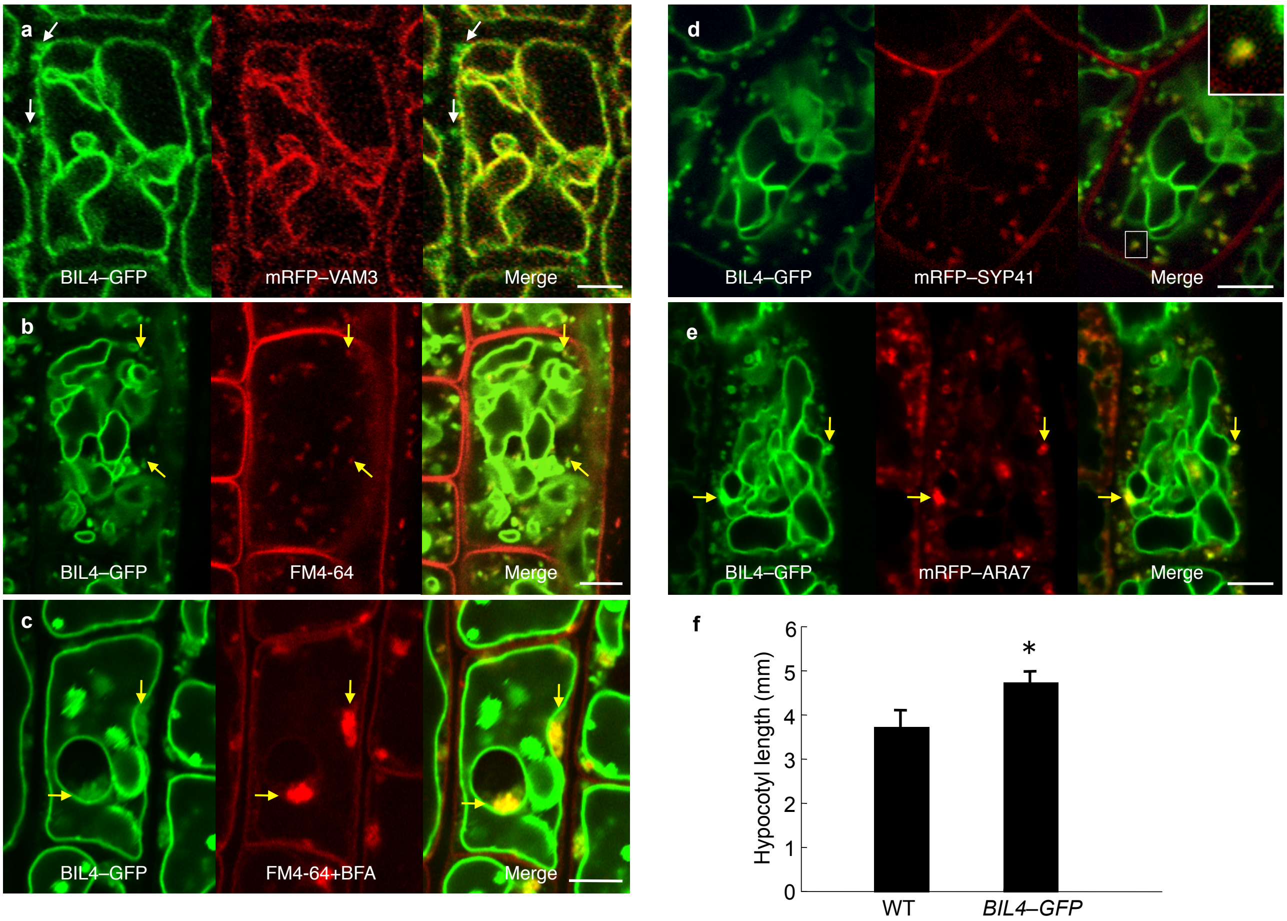

Figure S9 | 35S::BIL4–GFP localizes to punctate structures and the vacuolar membrane. (**a**) 35S::BIL4–GFP partially colocalizes with the vacuolar membrane marker mRFP–VAM3 (unmerged puncta are marked by white arrows). (**b**) 35S::BIL4–GFP partially colocalizes with the endocytic tracer FM4-64 (marked by yellow arrows). The seedlings were treated with FM4-64 for 5 min and then incubated in water for 20 min before imaging. (**c**) The endosomal compartment of 35S::BIL4–GFP and FM4-64 localization is sensitive to BFA. The seedlings were pretreated with FM4-64 for 5 min and then treated with 50 µM BFA for 30 min before imaging. The BFA compartments (marked by yellow arrows) containing 35S::BIL4–GFP and FM4-64 are merged. (**d**) 35S–BIL4–GFP partially localizes with the TGN/EE marker mRFP–SYP41. The inset at upper right shows the higher magnification of a punctate structure outlined with a rectangle. (**e**) 35S::BIL4–GFP partially colocalizes with the LE/MVB marker mRFP–ARA7 (marked by yellow arrows). Scale bars, 5 µm. (**f**) The BIL4–GFP protein functions in hypocotyl elongation. Hypocotyl lengths of wild-type and 35S::BIL4–GFP seedlings grown in the dark for 7 days on medium containing 3 µM Brz. The results are presented as the mean ± s.d., n > 26 seedlings. The asterisks indicate significant difference relative to wild-type plants ($P < 0.01$ by Student's *t*-test).

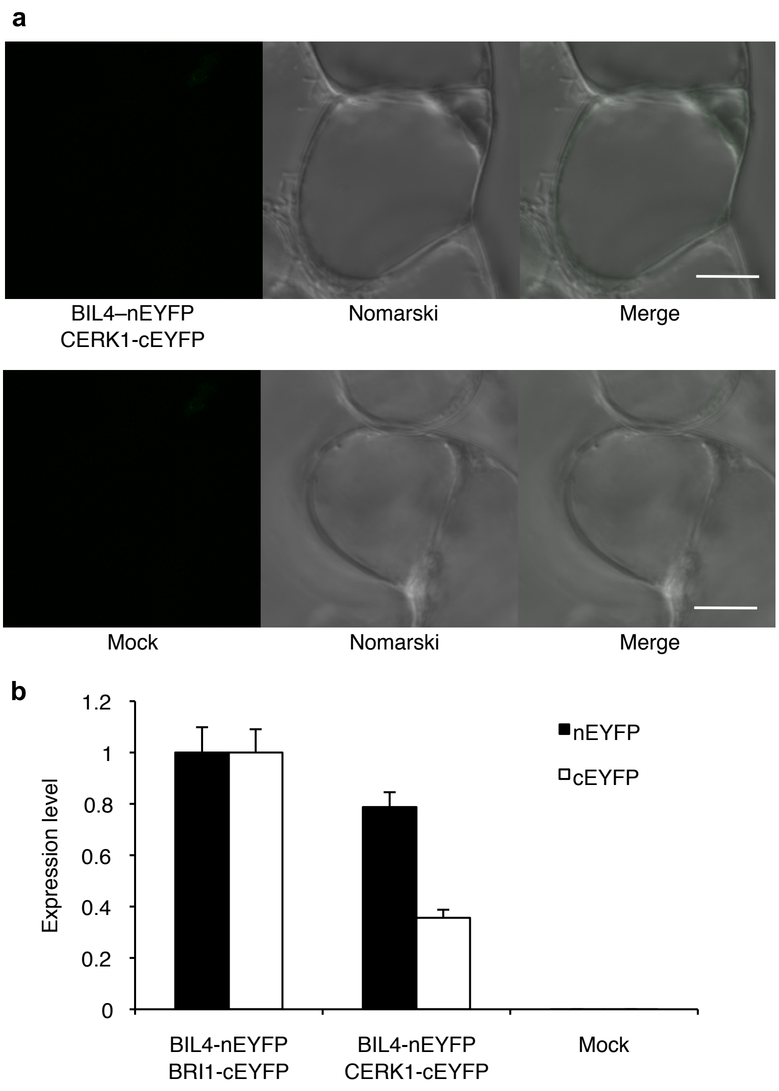

Figure S10 | Negative controls for the BiFC assay. (**a**) Cultured *Arabidopsis* cells were co-transformed with the BiFC constructs used in Figure 5g with CERK1–cEYFP. The GFP channels of confocal pictures acquired with the imaging settings that were used for Figure 5g are presented. Scale bars, 10 µm. (**b**) Quantitative RT-PCR analyses of the expression of cEYFP and nEYFP in the BiFC assay sample presented in Fig. 5g and (**a**). The results are presented as the mean ± s.d.

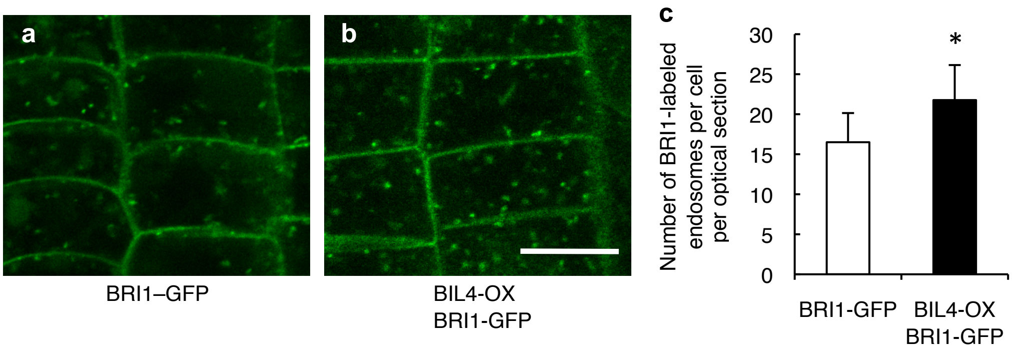

Figure S11 | Number of BRI1–GFP-labeled endosomes increased in the *BIL4-OX* plants as compared to the wild-type plants. (**a**,**b**) BRI1-GFP-labeled endosomes in the wild type (a) and *BIL4-OX* (b). Scale bar, 10 µm. (**c**) Number of BRI1-labeled endosomes per cell per optical section of root cells of four-day-old seedlings. n=30 cells. *P<0.01, Student's t-test. The results are presented as the mean ± s.d.

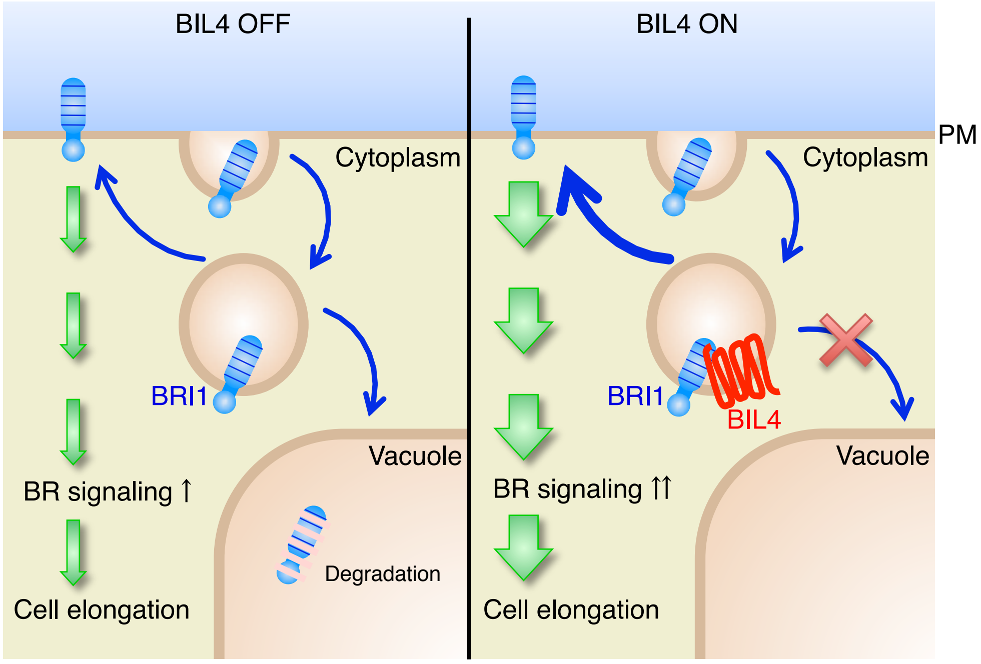

Figure S12 | A model illustrating the contribution of BIL4 to the BR signaling pathway controlling cell elongation. BRI1 accepts BR on the plasma membrane and activates BR signaling. BRI1 cycles between the plasma membrane and endosome and is sorted from the endosome to the vacuole for degradation through the endosome. In the presence of BIL4, BRI1 is internalized in the cell and colocalizes with BIL4 in the TGN/EE and LE/MVB. BIL4 inhibits BRI1 trafficking from the endosome to the vacuole and might promote the recycling of BRI1 to plasma membranes. Plasma membrane-localized BRI1 may activate a known BR signaling pathway and induce cell elongation.

Table S1 | List of the homologs of *BIL4* gene

Gene name	Accession number
Populus-1	XP_002307607.1
Populus-2	XP_002300819.1
Populus-3	XP_002307606.1
Populus-4	XP_002312548.1
Populus-5	XP_002312729.1
Populus-6	XP_002314428.1
Vitis-1	XP_002280313.1
Vitis-2	XP_002524728.1
Vitis-3	XP_002283304.1
Vitis-4	CBI26161.3
G. max-1	ACU16623.1
G. max-2	ACU14127.1
Rice-1	NP_001059026.1
Rice-2	NP_001051551.1
Rice-3	NP_001051246.2
Rice-4	NP_001068154.1
Rice-5	NP_001051550.1
Rice-6	NP_001059025.2
Rice-7	NP_001055493.1
Z. mays-1	NP_001130584.1
Z. mays-2	NP_001149807.1
Z. mays-3	NP_001151352.1
Z. mays-4	NP_001136515.1
Z. mays-5	NP_001149877.1

Gene name	Accession number
Picea-1	ABR16790.1
Picea-2	ABK27003.1
Picea-3	ABK25077.1
Ricinus	XP_002516809.1
P. patens-1	XP_001757037.1
P. patens-2	XP_001768467.1
P. patens-3	XP_001768622.1
Selaginella-1	XP_002975127.1
Selaginella-2	XP_002968030.1
Selaginella-3	XP_002971406.1
Chlorella	EFN57628.1
Ectocarpus	CBJ28269.1
zebrafish	NP_998303.2
zebra finch	ACH43670.1
Horse	XP_001491162.1
Rattus	NP_954547.1
Mouse	NP_080893.1
Human	AAF14868.1
Camelpox virus	NP_570396.1
Vaccinia virus	AAW21698.1
Culex	XP_001865933.1
Drosophila	NP_523722.1
Nectria	XP_003046723.1
Coccidioides	XP_001242790.1
Botryotinia	XP_001551993.1
Arthroderma	XP_003012882.1
S. pombe	NP_588431.1
Coprinopsis	XP_001830726.1

Table S2

Endogenous BR profiles of wild-type and *BIL4-OX* for each independently grown biological replicate

	replicate 1		replicate 2		replicate 3	
BR intermediates	Col	*BIL4-OX*	Col	*BIL4-OX*	Col	*BIL4-OX*
6-DeoxoCT	0.63	0.94	0.62	0.48	0.29	0.99
6-DeoxoTE	0.05	0.06	0.02	0.01	0.02	0.02
6-Deoxo3DT	0.35	0.33	0.39	0.33	0.35	0.17
6-DeoxoTY	2.55	2.20	2.47	1.9	2.63	2.57
6-DeoxoCS	4.47	3.35	4.21	3.13	4.24	3.38
CT	nd	nd	nd	nd	nd	nd
TE	nd	nd	nd	nd	nd	nd
3DT	nd	nd	nd	nd	nd	nd
TY	0.34	0.20	0.31	0.18	0.28	0.24
CS	0.48	0.33	0.51	0.31	0.49	0.38
BL	nd	nd	nd	nd	nd	nd

BIL4-OX partially decreases the level of BR intermediates downstream of 6-Deoxo3DT. The endogenous levels are shown in ng/g fresh weight. 6-DeoxoCT, 6-deoxocathasterone; 6-DeoxoTE, 6-deoxoteasterone; 6-Deoxo3DT, 3-dehydro-6-deoxoteasterone; 6-DeoxoTY, 6-deoxotyphasterol; 6-DeoxoCS, 6-deoxocastasterone; CT, cathasterone; TE, teasterone; 3DT, 3-dehydroteaserone; TY, typhasterol; CS, castasterone; BL, brassinolide. nd, not detected.
